# The Complex Role of Lactic Acid Bacteria in Food Detoxification

**DOI:** 10.3390/nu14102038

**Published:** 2022-05-12

**Authors:** Penka Petrova, Alexander Arsov, Flora Tsvetanova, Tsvetomila Parvanova-Mancheva, Evgenia Vasileva, Lidia Tsigoriyna, Kaloyan Petrov

**Affiliations:** 1 Institute of Microbiology, Bulgarian Academy of Sciences, 1113 Sofia, Bulgaria; pepipetrova@yahoo.com (P.P.); alexander_arsov@abv.bg (A.A.); 2 Institute of Chemical Engineering, Bulgarian Academy of Sciences, 1113 Sofia, Bulgaria; florablue@abv.bg (F.T.); mila_parvanova@abv.bg (T.P.-M.); jenivasileva96@gmail.com (E.V.); lidinka29@gmail.com (L.T.)

**Keywords:** food, lactic acid bacteria, toxins, mycotoxins, pesticides, heavy metals, antinutrients

## Abstract

Toxic ingredients in food can lead to serious food-related diseases. Such compounds are bacterial toxins (Shiga-toxin, listeriolysin, Botulinum toxin), mycotoxins (aflatoxin, ochratoxin, zearalenone, fumonisin), pesticides of different classes (organochlorine, organophosphate, synthetic pyrethroids), heavy metals, and natural antinutrients such as phytates, oxalates, and cyanide-generating glycosides. The generally regarded safe (GRAS) status and long history of lactic acid bacteria (LAB) as essential ingredients of fermented foods and probiotics make them a major biological tool against a great variety of food-related toxins. This state-of-the-art review aims to summarize and discuss the data revealing the involvement of LAB in the detoxification of foods from hazardous agents of microbial and chemical nature. It is focused on the specific properties that allow LAB to counteract toxins and destroy them, as well as on the mechanisms of microbial antagonism toward toxigenic producers. Toxins of microbial origin are either adsorbed or degraded, toxic chemicals are hydrolyzed and then used as a carbon source, while heavy metals are bound and accumulated. Based on these comprehensive data, the prospects for developing new combinations of probiotic starters for food detoxification are considered.

## 1. Introduction

In addition to nutrients, human food sometimes contains components and ingredients of a toxic nature. Food poisoning and foodborne illness outbreaks have been a problem for human communities since the dawn of civilization. Such data go back to antiquity when the population of ancient Rome used lead pipes to build aqueducts and sweetened the wine with lead acetate (Pb(C_2_H_3_O_2_)_2_·3H_2_O), known as lead sugar. The Middle Ages in Europe were marked by numerous incidents of human poisoning after eating rye-flour bread infected with ergot fungi. The types, severity, and consequences of food-related diseases have changed over the centuries and remain diverse in different regions and communities. In the last two decades, we have witnessed the deadliest outbreaks caused by toxigenic microorganisms. *Listeria monocytogenes* struck South Africa in 2017, poisoning 1060 people and killing 216; new toxigenic strain *E. coli* O104:H4 caused 53 deaths and serious illness of more than 3950 people in Europe in 2011; aflatoxin contamination of maize in Kenya resulted in 317 cases of hepatic failure and 125 deaths in 2004 [1,2]. In terms of chemical contamination, a significant incident occurred in China in 2008, when infant milk formula was contaminated with melamine, resulting in 294,000 affected babies, 6 of whom died [3].

Today, more than 200 diseases are caused by eating food contaminated with bacteria, viruses, parasites, toxins, or chemicals. This contributes significantly to the global increase in morbidity and mortality. Worldwide, about 600 million people get sick each year from eating contaminated food, which leads to 420,000 deaths annually, mostly of children and vulnerable people [4]. That is why the WHO established the Foodborne Disease Burden Epidemiology Reference Group (FERG). According to the report, food poisoning has exerted a significant socio-economic impact and emerged as a growing public health problem in the last decade. The Secretariat of the International Food Safety Authorities Network (INFOSAN) reported that only during the fourth quarter of 2021, 64 food safety incidents of great importance, involving 86 countries, occurred. Thirty-three of them posed a serious biological hazard to society and were caused by toxigenic *Salmonella* spp., *Lis. monocytogenes*, *E. coli*, *Bacillus cereus*, *Vibrio* spp., *Clostridium botulinum*, *Staphylococcus aureus*, and *Shigella sonnei* [4].

However, food containing chemical agents does not pose a lesser risk to the health of consumers. Notably, 140,000 tons of pesticides are sprayed on crops in the European Union every year [5]. The toxicological evaluation of pesticide residues in food performed by the Food and Agriculture Organization (FAO) showed that the residual concentration of 13 toxic pesticides must be continuously monitored because they leave significant traces in food commodities [5].

**Figure 1:**
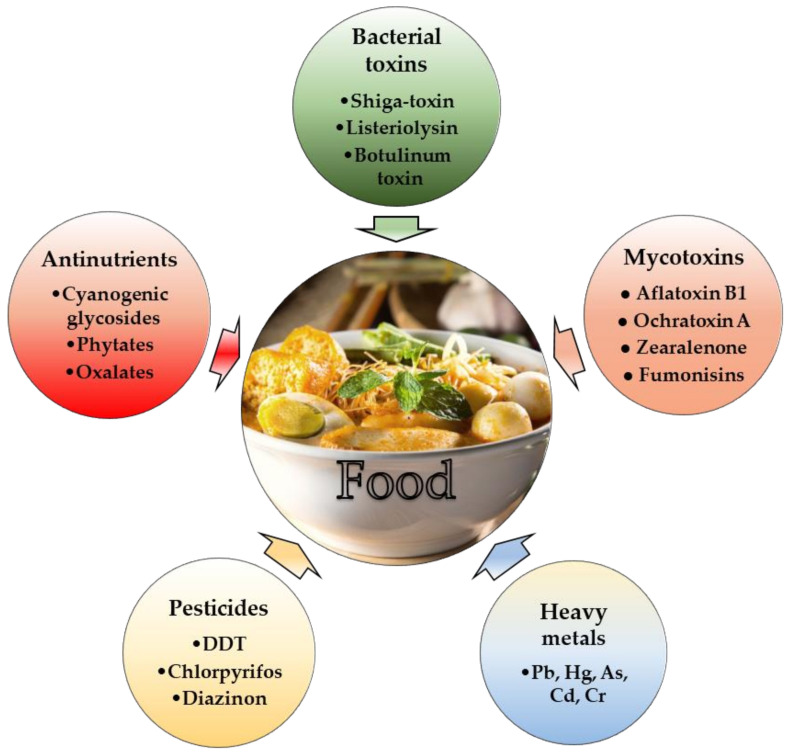
Scheme of the toxic compounds that could be found in food products.

According to the World Health Organization (WHO), toxic ingredients in food may be classified as (i) toxicants derived from microorganisms; (ii) toxic chemicals (pesticides, heavy metals); and (iii) naturally occurring toxicants and antinutrients derived from plant material before processing. All these agents cause gastrointestinal tract (GIT) disorders and inflict considerable neurological, cardiovascular, immunological, and psychological damage. A schematic overview of toxic food ingredients is shown in Figure 1.

Food spoilage can occur at various stages of food production, supply, and consumption. That is why food safety receives a lot of attention in wealthier societies, but it is a much more pressing concern in developing countries. One reason for food contamination is the polluted water used for washing and processing; others include primitive ways of production and improper use of agricultural chemicals, poor storage, and lack of regulations. Many agents that cause diseases are transmitted from domestic animals to humans through food products; in addition, the warm climate further contributes to the spread of natural toxigenic producers in tropical countries.

Lactic acid fermentation is the oldest and most widely used method to improve the safety and nutritional value of foods. It has been employed from the very beginning of agriculture and animal husbandry to preserve cereal, milk, fish, and meat products from bacterial contamination, prolong their shelf life, and enrich them with probiotic bacterial strains [6,7]. Lactic acid bacteria (LAB) are routinely used to produce traditional functional foods such as yogurt, cheese, sauerkraut, pickles, and fermented cereal meals and beverages [6,7,8]. Dozens of LAB strains have been evaluated as probiotics due to the production of metabolites with health benefits that are scientifically confirmed and well-documented [9,10,11,12]. However, Markowiak and Śliżewska have underlined that one of the requirements for a particular strain to be evaluated as a probiotic should be its ability to inhibit the production of bacterial toxins, inactivate them, or facilitate their removal from the human body [13]. The probiotics exhibiting detoxifying properties contain unique, strain-related characteristics, and their selection deserves special attention. On the other hand, over the past decade, hundreds of scientific studies have highlighted the role of LAB in food detoxification [14,15,16]. Large-scale food production and increasing environmental pollution make the topic of natural food purification via microbial fermentation extremely important and relevant. Biological detoxification of food can be achieved with various LAB degrading, metabolizing, or adsorbing toxins and thus effectively neutralizing them. The present state-of-the-art review aims to summarize the available data and elucidate the current role of LAB in food detoxification. Due to their wide substrate spectrum and diverse enzyme pool [17,18,19], LAB can ferment almost any food of dubious quality and potentially detoxify it. The unique properties of LAB that make them the “panacea” for food detoxification are described below.

## 2. Lactic Acid Bacteria as Probiotics

Although the original concept of probiotics was first proposed more than a hundred years ago by Élie Metchnikoff, the term was introduced in 1965 by Lilly and Stillwell to describe the consumption of a living microorganism with a positive effect on the resident microflora [20]. In 2010, Fujiya and Kohgo widened the definition by including other positive effects on human health, such as “maintaining intestinal development, nutrition and treatment of intestinal inflammation, functional disorders and other extraintestinal diseases” [21]. Indeed, besides the ability to maintain the proper balance between pathogens and the beneficial bacteria in order to prevent gastrointestinal infections and disorders [22], probiotics also possess immunomodulatory action on the host [23], alleviate allergies and atopic diseases [24], and help in cholesterol removal [25].

The most significant share of probiotic microorganisms is occupied by LAB species of the genus *Lactobacillus* as well as the species *Enterococcus faecalis* and *Ent. faecium*, *Lactococcus lactis*, *Leuconostoc mesenteroides*, *Pediococcus acidilactici*, *Sporolactobacillus inulinus*, and *Streptococcus thermophilus* [26]. Among lactobacilli, the most popular pharmaceutical probiotics contain *Lactobacillus acidophilus*, *Lacticaseibacillus casei*, *Lactobacillus gasseri*, *Limosilactobacillus reuteri*, and *Lactobacillus helveticus*, while most often used in the production of functional foods are the species *L. amylovorus*, *Lactiplantibacillus plantarum*, *Lacticaseibacillus paracasei*, *Lactobacillus johnsonii*, *Lactiplantibacillus pentosus*, and *Lactiplantibacillus rhamnosus* [13].

**Figure 2:**
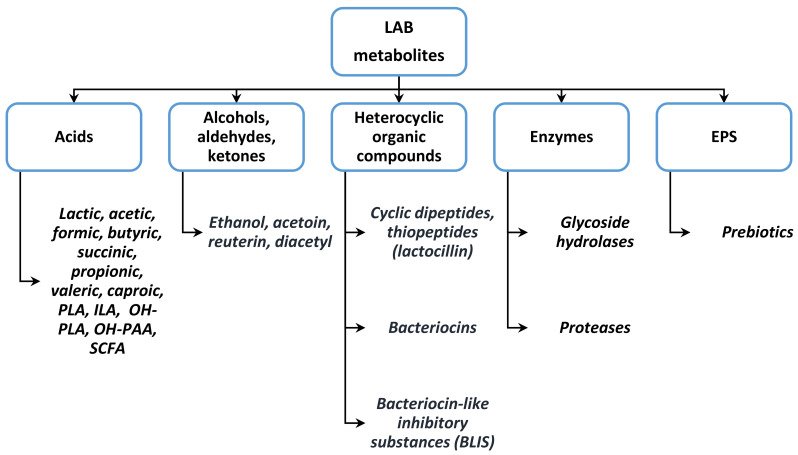
LAB metabolites involved in their activity against toxigenic producers. Designations: PLA, phenyllactic acid; ILA, indolelactic acid; OH-PAA, hydroxyphenylacetic acid; OH-PLA, 4-hydroxyphenyllactic acid; SCFA, branched short-chain fatty acids; EPS, exopolysaccharides.

The main LAB metabolites that may be used against toxigenic producers are shown in Figure 2. Lactic acid (LA) has a well-established antimicrobial activity. According to Arena et al. [27], LA acts in its protonated form by impairing the pH gradient between the cytosol (alkaline) and the external environment (acidic), thus dissipating the membrane potential and destroying the pathogenic cells. Birt et al. [28] showed that other organic acids with antimicrobial effects are branched short-chain fatty acids (SCFA) such as isobutyrate and isovalerate. Fazeli et al. [29] reported the same effect in the production of acetate, butyrate, formate, succinate, propionate, valerate, and caproic acid.

Van der Meulen et al. [30] showed that LAB also form phenyllactic, indolelactic, 4-hydroxyphenyllactic, and hydroxyphenylacetic acids, all possessing antimicrobial activity in sourdough, while Negatu et al. [31] noted the fungus-inhibitory activity of benzoic acid, methylhydantoin, and mevalonolactone. Indole-3-propionic prevents endotoxins leakage through the intestinal epithelial barrier [32]. Other antimicrobial compounds are carbonyl derivatives such as diacetyl, acetaldehyde, acetoin, and 2,3-butanediol, as they act against toxigenic *E. coli*, *Lis*. *monocytogenes*, and *S. aureus* [33]. Carbon dioxide works by inhibiting enzymatic decarboxylation and increasing the membrane permeability, while hydrogen peroxide damages cellular structures through its oxidative effect and disrupts the membrane redox potential [34].

Bacteriocins are ribosomally produced, heat- and acid-resistant LAB oligopeptides with antimicrobial activity against foodborne pathogenic bacteria and fungi. According to the nucleotide sequence of the responsible genes and amino acid composition, bacteriocins are classified as (1) smaller than 5 kDa, heat-stable, and lanthionine-containing; (2) bacteriocins below 10 kDa, heat-stable, and non-lanthionine-containing; (3) proteins with Mw higher than 30 kDa, heat-sensitive; and (4) bacteriolysins. Due to their efficiency, class II (pediocin-like bacteriocins) are considered an alternative to chemical preservatives because they are highly active against *Lis. monocytogenes*. The specific structure of these molecules includes conserved Tyrosine-Glycine-Asparagine-Glycine-Valine (YGNGV) motif and disulfide bonds in the N-terminal region [35]. Bacteriocins with a pronounced antitoxigenic activity belong to the third class: helveticin M, helveticin J, and enterolysin A, produced by *Lactobacillus crispatus*, *L. helveticus*, and *Ent. faecalis*, while in the fourth class are affiliated with leuconocin S and lactocin 27, which comprise complexes of protein, lipids, and carbohydrates. One of the most active antibacterial compounds, plantaricin A, is produced by many food-derived LAB: *Lp. plantarum*, *Furfurilactobacillus rossiae*, *Levilactobacillus brevis*, *Companibacillus paralimentarius, Leuc. mesenteroides, Leuc. pseudomesenteroides*, and *Leuc. citreum*; *Weissella paramesenteroides*, *W. cibaria*, *Lactiplantibacillus paraplantarum*, and *Latilactobacillus curvatus* [36]. Other bacteriocins secreted by food LAB are sakacin (*Leuc. citreum* and *Latilactobacillus graminis*), bavaricin (*Latilactobacillus sakei*), and pentocin. *L. gasseri* produces gassericin A and the thiopeptide antibiotic lactocillin, which prevents the growth of *Staph. aureus*, *Corynebacterium aurimucosum*, and *Str. sobrinus* [37].

Other LAB metabolites with strong antifungal activity are cyclic dipeptides. They are often produced by sourdough lactobacilli species such as *Fur. rossiae, L. harbinensis, L. amylovorus*, *Limosilactobacillus reuteri*, *Lev. brevis*, and *Levilactobacillus spicheri* [38]. Different *Lp. plantarum* strains generate the fungistatic peptides cyclo(Gly-L-Leu), cyclo(L-Phe–L-Pro), cyclo(L-Phe–trans-4-OH-L-Pro), cyclo(L-Leu-L-Pro), and cyclo(L-Phe-L-Pro) [31,39,40]. Other antifungal cyclopeptides produced by sourdough LAB are cyclo(L-Pro-L-Pro) [41], cyclo(L-Tyr-L-Pro) [42], cyclo(L-Met-L-Pro) [43], cyclo(L-His-L-Pro) [44], and cyclo(Leu-Leu) [45].

## 3. LAB against Bacterial Toxins and Their Producers

There are three types of bacterial foodborne diseases: intoxications, infections, and toxico-infections. Intoxication occurs by ingesting food containing a pre-formed bacterial toxin (for example, produced by *S. aureus* or *C. botulinum*), which causes intoxication. The second type of foodborne infection is a result of the consumption of food containing viable toxigenic bacteria (such as serotypes of *E. coli*, *Salmonella* or *Listeria*), which multiply in the host and cause disease. The third variant (toxico-infection) is caused by species such as *C. perfringens*: When food containing viable vegetative cells is consumed, bacterial cells undergo sporulation in the small intestine and produce an enterotoxin, which is released with the spores during cell lysis. The enterotoxin has a cytotoxic effect on GIT epithelial cells by damaging the cell membrane structure.

### 3.1. LAB against Toxigenic Escherichia coli

Many authors studying spontaneously fermented ethnic foods believe that the presence of probiotics in the diet can serve as a preventive measure against infectious diseases associated with the consumption of contaminated foods [7]. However, besides enteritis and diarrhea, the toxigenic *E. coli* strains can cause urinary tract infections, septicemia, neonatal meningitis, and cardiovascular and central nervous system diseases by cytotoxin production and are the most clinically significant pathogen in European countries [46]. Shiga toxin-producing *E. coli* (STEC) are enterohemorrhagic *E. coli* (EHEC) strains that cause either enteric disease (bloody diarrhea, hemorrhagic colitis) or hemolytic uremic syndrome (HUS). EHEC colonizes the host’s large intestine, causing the so-called attaching-and-effacing (AE) lesions. The adherence to epithelial cells with localized destruction occurs with the aid of Shiga toxins 1 and 2 (Stx1, Stx2). These two cytotoxins are immunologically different, as Stx1 is identical to the Shiga toxin produced by *Shigella dysenteriae* type I [47]. Both toxins are encoded on chromosomal lysogenic bacteriophages. Although many variations are found in the Stx family, all Shiga toxins have an A-B subunit structure. Subunit A has N-glycosidase activity, while subunit B binds to a membrane glycolipid. Subunit A cleaves a single adenine residue from the 28S rRNA component of eukaryotic ribosomes, resulting in inhibition of protein synthesis in the cells of the renal glomeruli [48].

STEC infections can lead to death, especially in young children and elderly people. Various foods contain such strains: ground beef, fresh milk, apple cider [49,50,51,52,53,54,55], or fermented hard salami [56]. Although the most dangerous EHEC, *E. coli* O157:H7, has been associated with foods of bovine origin in Michigan and Oregon, USA, in 1982 [57], it was also found in goat’s milk, lettuce, and alfalfa sprouts [58,59,60,61]. Investigating the presence of STEC in 4330 Korean food samples, Ryu et al. [62] determined the highest prevalence of the bacterium in yukhoe (forged raw meat), cold bean soup, gimbal (meat broth for cold noodles), and sprouts, as well as that 17.7% of the obtained *E. coli* strains, were resistant to antibiotics. A study was also conducted in central Egypt to determine whether *E. coli* O157:H7 was present in 175 samples of raw ground beef, chicken, lamb, and unpasteurized milk obtained from slaughterhouses, supermarkets, and farms [63]. In Greece, 1–2% of samples of ewes’ milk, sausages, and swine intestines contained *E. coli* O157:H7; similar values were obtained in the Czech Republic and Spain [64,65,66]. STEC/enteroaggregative *E. coli* O104:H4 was the causative agent of the outbreak that occurred in Germany in 2011 and took at least 40 lives from more than 4000 cases of diarrhea; almost one-fourth of the cases (908) and more than 75 percent of the deaths (34) were accompanied by hemolytic-uremic syndrome [67].

LAB can inhibit the growth of STEC/EHEC *E. coli* serotypes by direct or indirect interaction with the pathogen. For instance, Orihuel et al. [68] tested LAB antagonistic activity against STEC strains in co-cultures. Bacteriocin producer *L. curvatus* CRL705 showed only a slight decrease in the *E. coli* population, whereas the bacteriocinogenic strain *Ent. mundtii* CRL 35 and *Lp. plantarum* CRL 681 (non-bacteriocinogenic) significantly reduced *E. coli* viability and put its growth into the death phase after 8 h. In order to assess the antagonistic mechanisms, a proteomics approach was applied. Differences in the proteome were connected with carbohydrate and amino acid metabolism, energy production, transcription, and translation; cell division was also involved, suggesting that *Ent. mundtii* CRL35 used the competition strategy.

The inhibitory characteristics of probiotic strains *Lc. casei* Shirota and *L. acidophilus* YIT 0070 were investigated toward three clinical isolates of *E. coli* O157:H7. During batch co-fermentation, both probiotic lactobacilli exerted growth inhibitory and bactericidal activity on EHEC [69]. The same authors, Ogawa et al. [70], used a newborn rabbit model of experimental infection to investigate the protective effects of oral administration of the probiotic *Lc. casei* strain Shirota against EHEC infection. Daily consumption of the probiotic from birth prevented colonization in the GIT and reduced the concentrations of both Stx1 and Stx2 toxins. The reason for the protective effect of *Lc. casei* Shirota was due to local immune response enhancement and STEC cell elimination, which consequently reduced toxin levels in the gut. Byakika et al. [71] revealed the antimicrobial effect of *Lp. plantarum*, *Lactococcus lactis*, *W. confusa*, and *Lc. rhamnosus* GG against acid- and antibiotic-resistant *E. coli* producing Stx2 toxin and isolated from Obushera, a Ugandan cereal drink. The data concerning the molecular mechanisms involved in the antimicrobial activity of LAB against STEC and EHEC are summarized in Table 1.

Several different studies claimed that the popular probiotic *L. acidophilus* strain La-5 is effective against infection with toxigenic *E. coli* O157:H7. Zeinhom et al. [77] observed an antivirulence effect of an “active fraction” extracted from La-5 cell-free spent medium incorporated in yogurt and tested using a mice model. Strain-derived metabolites prevented the epithelium attachment and GIT colonization by STEC, along with crucial downregulation of the *stxB2* gene encoding Shiga toxin. This study confirmed earlier works with the same probiotic, which reported the ability of *L. acidophilus* strain La-5 to prevent EHEC from adhering to epithelial cells and to concentrate F-actin at adhesion sites [78]. The surface-layer protein (SLP) extracts of *L. helveticus* and *Lc. rhamnosus* metabolites decrease the AE lesions of *E. coli* O157:H7. Both species preserve the barrier function of Hep-2 and T84 cells monolayers by metabolites produced in the culture medium [79]. Hirano et al. [80] also found that *Lc. rhamnosus* prevents EHEC adhesion to human colon epithelial cell line C2Bbe1, but only when living probiotic cells are used.

*Lc. rhamnosus* GR-1 and *Li. reuteri* RC-14 were studied for their effects on growth and virulence expression factors in uropathogenic *E. coli* C1212. LA and other metabolites secreted by lactobacilli downregulate genes for proteins critical for the pathogen’s attachment [81]. Caridi et al. [82] evaluated *Lc. paracasei* subsp. *paracasei* isolated from Italian cheese as *E. coli* antagonist due to bacteriocin production. A recent study by Fijan et al. [83] revealed the high potential of *Li. reuteri* DSM 17,938 to diminish EHEC growth; however, the authors admitted that the most effective antagonism against EHEC was displayed by multi-strain culture containing lactobacilli, bifidobacteria, and enterococci.

### 3.2. LAB against Listeria Monocytogenes

*Lis. monocytogenes* is a non-spore-forming opportunistic pathogen, an intracellular parasite expressing β–hemolysin [84,85]. In nature, it grows in soil, water, and plant material. This pathogen causes listeriosis, characterized by central nervous system disorders, mainly meningitis and encephalitis, pneumonia, respiratory problems, and hematologic deviations [86]. Susceptible to listeriosis are immunocompromised individuals, pregnant women, newborns, and the elderly, as 20–30% of the infected people reach a lethal end [87]. If untreated during pregnancy, the illness could lead to amnionitis and fetus infection, premature birth, or abortion [88]. The responsible factor for this severe infection is a listerial toxin, listeriolysin O (LLO), accompanied by transcriptional activator (PrfA), actin (ActA), and surface proteins internalins InlA and InlB. The presence of responsible genes in food is evidence of *Listeria* infection [89]. LLO is a cytolysin that is activated by reducing agents (thiol groups), with maximal cytolytic activity at pH 5.5 and 37 °C. The toxin is activated in the phagosomes and lyses them, thus allowing *Lis. monocytogenes* to escape into the cytosol and persist intracellularly, protected from the immune system [90].

The basic way of introducing foodborne pathogens into the human organism is via food products—most often the so-called ready-to-eat food. *Lis. monocytogenes* can be found in fruits, vegetables, meat, poultry, raw milk and dairy products, and seafood [91]. Among fresh products, *Lis. monocytogenes* is found to grow on cabbage, potatoes, asparagus, green beans, broccoli, radishes, corn, cauliflower, lettuce [90], in refrigerated and cooked eggs [92], and cantaloupe [93]. Besides food, strains of *Lis. monocytogenes* can also contaminate non-food contact surfaces, such as sinks and grounds, persisting for a long period even without growth [94]. It can survive in critical conditions—temperatures between −0.5 °C and 45 °C, high osmotic pressure up to 10% NaCl, and low pH values such as 3.3–4.2 [95,96,97]. The pathogen adapts to stress conditions by altering its membrane fluidity [98], synthesizing σ-factors and osmoprotectant molecules—proline, glycine, betaine, acylcarnitine, and carnitine [99].

Traditional approaches against foodborne infections with *Lis. monocytogenes* include heating, salting, acid treatment, and drying [100]. Modern technologies include the application of high hydrostatic pressure, pulsed electric field, new packaging methods, and biocontrol. With the latter, the environmental method, LAB metabolites are involved [101]. LAB counteract toxigenic strains of *Listeria* with all available antimicrobial agents, the most effective against this pathogen being organic acids and bacteriocins. The biocontrol is accomplished by two methods which introduce bacteriocins into the food. In the direct approach, the bacteriocin is added in the form of concentrated dried powder. In the indirect method, bacteriocin-producing LAB strains are incorporated into the food and secrete bacteriocins in situ. In order to prevent the decrease in activity over time because of enzymatic degradation, interference with food components, or food processing, methods for the inclusion of bacteriocin in structures consisting of alginate, gelatin, starch, guar gum, xanthan gum, or liposomes are developed [102]. The most promising LAB strains in the fight against *Lis. monocytogenes* and the main tools they use are listed in Table 2.

LAB, which produce bacteriocins with anti-listerial activity, belong to the genera *Lactococcus*, *Lactobacillus*, *Leuconostoc*, *Enterococcus*, *Pediococcus*, and *Carnobacterium* [119]. The most studied bacteriocins with bacteriostatic activity are nisin produced by some *Lactococcus lactis* spp. *lactis*; pediocin—by *Pediococcus* spp. [108]; and plantaricin—by *Lp. plantarum*. Bavaricin A has a bactericidal mode of action on 90% of the tested *Lis. monocytogenes* strains [36]. It is produced by the sourdough strain *L. bavaricus* MI401. Similar to nisin, it is synthesized at temperatures from 4 °C to 30 °C. Another sourdough isolate, *Fructilactobacillus sanfranciscensis* strain C57, produces a chromosomally-encoded bacteriocin-like inhibitory substance (BLIS) active against the same pathogen. Nisin is approved as a legal food additive in many countries [89]. Some successful encapsulations of nisin in soy-lecithin are available in the literature [120,121]; however, the non-encapsulated one demonstrates stronger anti-listerial activity [122]. According to Thomas and Wimpenny [123], nisin activity increases with the decrease in temperature and pH. For achieving enhanced nisin activity against foodborne pathogens, combinations of nisin with other compounds have been applied. For example, the product Nisaplin^®^ consists of nisin (2.5% *w/w*), NaCl (77.5% *w/w*), protein (12% *w/w*), and carbohydrates (6% *w/w*) [89]. Notably, LAB inhibit *Lis. monocytogenes* in food products under refrigerating temperatures. Amezquita and Brashears [109] registered strong anti-listerial activity of *P. acidilactici*, *Lc. casei*, and *Lc. paracasei* at 5 °C isolated from ready-to-eat foods. Even higher activity is observed in cases of co-cultivation of different LAB strains in combination with ProH (whey protein hydrolyzed with pepsin) in traditional Spanish cheese [124]. Morandi et al. [125] achieved total inhibition of the pathogen throughout the co-cultivation of *Lactococcus lactis* FT27 and *Carnobacterium divergens* SCA, inoculated in Gorgonzola cheese, and the addition of lactic acid/sodium lactate.

Another strategy for *Lis. monocytogenes* prevention is the potential of LAB to be employed as a vaccine vector. The LLO possesses important features, enhancing its potential in antitumor vaccines, such as the ability to live intracellularly in a host cell that is not infected by other toxin-producing bacteria [126] and the ability to provide cytosolic access for antigens in antigen-presenting cells via pores formation [127]. As the infection occurs through contaminated food and the pathogen succeeds in bypassing the mucosal barrier, the mucosal vaccines would offer higher effectiveness than those with a parenteral delivery route [128]. However, antigen delivered by mucosa leads to a weak immune response, most likely due to fast disruption in the mucosal secretion, low microbial adsorption, and mucosal tolerance [129]. The safe oral uptake of LAB makes them quite attractive to be employed as a live vector. In this regard, the most studied are the LAB exhibiting probiotic features [130]. *Lactococcus lactis* appears to be the most suitable for vaccine production as its safety is confirmed and its genome is completely sequenced [118]. Its capability to express different antigens intra- and extracellularly resulted in the development of an inducible expression system. This system should be used for listerial antigens expression delivered orally and be involved in the vaccine construction. LAB are also reported to demonstrate single-chain antibody fragments, which could be employed for generating passive immunity [131]. This is a possible strategy for *Lis. monocytogenes* treating, as it would exhibit a more direct and fast response. However, the questions about the horizontal transfer of plasmid carrying antibiotic resistance marker to the environmental and host microflora [118], the immune response regarding administration, and the rate of antigen production in vivo to stimulate future vaccine production based on the LAB system remain to be studied in more detail.

### 3.3. LAB Preventing the Growth and Toxin Production by Clostridium botulinum

*C. botulinum* is an obligately anaerobic, spore-forming microorganism, and first isolated from raw ham and human liver. Botulinum neurotoxins (BoNTs) are the most powerful natural toxins known to humankind [132]. They cause botulism, a rare but potentially fatal paralytic disease affecting both humans and animals. There are seven types of botulinum neurotoxins (A–G) and many subtypes (e.g., A1–A5 and several subtypes B, E, and F) with different amino acid sequences. BoNTs are initially formed as single-chain polypeptides with a molecular weight of about 150 kDa and relatively low toxicity. According to Lund and Peck [133], in the case of proteolytic *C. botulinum* (A, B, and F neurotoxins of Group I), the single-chain protein is cleaved by proteases to form a double-chained, highly toxic form. In non-proteolytic *C. botulinum* (B, E, and F type, Group II), the single-chain pre-toxin is not activated by the same proteases but by unidentified proteases in host cells. The responsible genes for the above-described groups of neurotoxins are either chromosomal or plasmid-located, while in groups III (C and D) and IV (G), neurotoxin genes are always plasmid-localized. Some strains contain genes for toxins of two different antigenic types, one synthesized in large quantities and the other in insignificant amounts. Although the vegetative cells of *C. botulinum* are sensitive to air, in spore form, they can retain viability for long periods. Spores of *C. botulinum* Group II pose the highest risk of food poisoning due to their ubiquitous presence in the environment and their ability to survive pasteurization [134], thus germinating in toxic cultures at low temperatures. Proteolytic strains can grow at temperatures below 10–12 °C (non-proteolytic—at 3–4 °C), and contaminate raw meat, fruits, vegetables, and seafood [135,136].

To prevent *C. botulinum* from spreading, many preservatives are used in food: 3.5% salt in the aqueous phase in chilled ready-to-eat foods, sodium or potassium nitrite and nitrate, etc. Many of these ingredients have detrimental effects on human health, mainly through the formation of carcinogenic substances such as nitrosamines [137].

Recently, the use of lactic acid bacteria was evaluated as a very effective approach to bio-control of *C. botulinum* [138]. The species that have been applied in solving this task until now are listed in Table 3.

There are various antimicrobials produced by LAB as part of their defense mechanisms that can improve their ability to compete with *C. botulinum*. Substances such as hydrogen peroxide, fatty acids, organic acids, ethanol, enzymes, and antibiotics are also involved in food defense against *C. botulinum* [144]. The use of bacteriocins in heat-treated foods can reduce the intensity of the heat process, minimize the cost of heat treatment and, at the same time, improve the nutritional and organoleptic properties of food [145]. Nisin effectively inhibits the growth of *C. botulinum* and its spores and prolongs the shelf life at room temperature [142]. To date, eight nisin types have been observed and characterized: Nisin A, Z, F, and Q are produced by *Lactococcus lactis*, while nisin U, U2, P, and H are produced by some strains of *Streptococcus* [146,147]. The concentration of 500–1000 IU/g nisin effectively inhibits *C. botulinum* in cheeses made from pasteurized milk [148,149]. Other LAB-derived bacteriocins, such as pediocin PA-1, mersacidin, mutacin, and lacticin, are used as preservatives in the food industry, as they are also able to prevent the growth of *C. botulinum*, *E. coli*, *Lis. monocytogenes*, *S. aureus*, and other food pathogens [150,151,152].

### 3.4. LAB Preventing the Growth and Toxin Production by Other Pathogenic Bacteria

Other widespread toxigenic foodborne pathogens are *C. perfringens*, *Bacillus cereus*, *S. aureus*, *Ps. fluorescens*, and *Ps. putida*. *C. perfringens* is a ubiquitous spore-forming bacterium, a contaminant of water and dust, but also foods such as meat and milk, even processed. The strains produce 18 different toxins and are classified into five toxin types (A, B, C, D, and E) according to the production of four major toxins (α, β, ε, and ι), and the sequences and localization of the toxin-encoding genes [153]. Five of the serotypes of the pathogen produce α-toxin, an enzyme of the family of bacterial zinc-metallo-phospholipases [154]. Both cells and cell-free supernatants of Chinese isolates of *L. acidophilus* and *Li. fermentum* inhibited the growth and α-toxin production by *C. perfringens*. In vitro experiments showed that both lactobacilli are able to degrade α-toxin [155].

*B. cereus* is another widespread food-spoiling and toxin-producing pathogen, the cause of many food-poisoning outbreaks. Its spores can be found in water, soil, air, cereals, rice, vegetables, milk, dairy products, and meat [156,157,158,159,160]. It is a common contaminant in raw milk, ice cream, milk powder, fermented milk, and pasteurized milk [161], as its spores are heat-resistant and survive pasteurization and chemicals. The ability of the bacterium to form biofilms makes it difficult to clean and disinfect. Once in the gastrointestinal tract, it causes two types of disease. Emetic syndrome is caused by the formation of heat-resistant emetic cereulide toxins (cyclic peptides), which the bacterium forms during its active phase of growth in food, and diarrhea syndrome, which is due to protein enterotoxin complexes, mainly hemolysin BL, non-hemolytic enterotoxin (NHE), and cytotoxin K produced during bacterial growth in the small intestine [162]. The clinical picture of ingestion of food contaminated with cereulide toxin includes nausea, vomiting and abdominal cramps appearing from the first to the fifth hour, and recovery is usually within 6–24 h [163]. The different strains of *B. cereus* have diverse pathogenic effects, with a dose for the diarrheal syndrome 10^5^–10^8^ CFU/g (colony-forming units per gram of food) of vegetative cells or spores, but there are exceptions, and food poisoning has also been reported with doses below 10^5^ CFU/g. Often two of the three enterotoxins work together and are responsible for gastrointestinal disorders by forming pores in the membranes of epithelial cells in the small intestine [164]. 

LAB act against *B. cereus* with the production of metabolites such as organic acids, hydrogen peroxide, bacteriocins, and other antimicrobial peptides [165]. Wang et al. [166] report that the antibacterial effect of LA is most likely due to physiological and morphological changes caused in the bacterial cytoplasmic membrane, leading to leakage of cytoplasmic content. In vacuum-packed raw meats and fish that are kept chilled, LAB become the dominant population and preserve the meat through so-called “hidden” fermentation. Tirloni et al. [167] report that the addition of natural microflora rich in lactic acid bacteria to yogurt, raw milk, and Taleggio cheese has led to inhibition of spore formation and subsequent development and growth of the vegetative cells of *B. cereus*. *L. acidophilus* LF221 and *Lactococcus lactis* have an enormous antibacterial activity against *B. cereus* in skim milk and fresh cheese due to the synthesis of lactic and acetic acids, while *Lacticaseibacillus paracasei* also prevents biofilm formation [167,168,169,170].

Besides *B. cereus*, LAB isolated from fermented foods display strong antagonism toward *S. aureus* and *Pseudomonas* spp., as reported by Olaniyi et al. [171]. *Ps. fluorescens* has generally been considered a saprophytic rhizobacterium; however, it has been isolated from human clinical samples and is known as a common contaminant of packaged vegetables, fish, chicken, beef, fruit milk, goat’s milk [172,173,174,175,176,177], as well as the raw milk in 28 different farms in the Lombardy region of Northern Italy in 2014 [178].

*Pseudomonas* spp. produce a large number of harmful extracellular substances: phytotoxic compounds, pigments, hydrocyanic acid, proteolytic enzymes, phospholipase, and several enterotoxins [179]. *Ps. fluorescens* also produces heat-resistant lipases and proteases, indigoidin (causing blue spots on mozzarella cheese), biosurfactants (in the chilled chicken meat), methyl mercaptan, and dimethyl disulfide in fish samples [174,179,180,181]. Exotoxins produced by *Pseudomonas* spp. are proteinaceous substances. When consumed with the food, they cause leukopenia, acidosis, circulatory collapse, liver necrosis, pulmonary edema, hemorrhage, and tubular necrosis of the kidneys, while proteolytic enzymes are responsible for hemorrhagic and necrotic changes in the skin, as well as corneal destruction in some eye infections [182]. LAB possess significant bactericidal activity against pseudomonads, as the main antagonistic tools are LA and bacteriocins. Among fifteen LAB isolated by Okorhi et al. [183], 80% showed antagonist activity against *Pseudomonas* spp., including *Lp. plantarum*, *Li. fermentum*, *L. acidophilus*, *Str. thermophilus* and *Lactococcus lactis*. Table 4 presents a summary of the most notable examples of antibacterial activity shown by LAB, including *Lc. paracasei* FX-6, which is highly effective against *Ps. putida* [184], and *Lc. rhamnosus*, which inhibits the formation of biofilm by the same pathogen [170].

*S. aureus* is a non-spore-forming opportunistic pathogen producing cytotoxins, exotoxins, and exfoliative toxins [186]. It causes many skin infections such as boils, pimples, cellulite and osteomyelitis, impetigo, and abscesses, as well as life-threatening diseases such as endocarditis, pneumonia, meningitis, and septicemia [187]. Staphylococcal food poisoning causing gastroenteritis is accompanied by symptoms of sudden onset of nausea, vomiting, abdominal cramps, and diarrhea, caused by ingestion and absorption of enterotoxins previously formed in food [188]. Up to half of the human population carries this bacterium; in addition, it grows in a wide pH and temperature range (pH 4.2 to 9.3, T °C to 48.5 °C), and up to 15% NaCl. However, LAB can minimize its spreading in food mainly by the action of lantibiotics produced by *Lactococcus lactis*. Felicio et al. [185] used nisin with concentrations of 400 and 500 IU/mL against the growth of *S. aureus* in Minas Frescal cheese and nisin-producing strain *Lactococcus lactis* UL730 against the enterotoxigenic *S. aureus* J10 in fresh Moroccan cheese. *Lp. plantarum* and *Lc. casei* active against *S. aureus* were isolated from Indian traditional fermented product dosa [189,190].

## 4. LAB against Mycotoxins and Their Producers

### 4.1. Mycotoxins—Overview and Medical Relevance

Mycotoxins are low-molecular secondary metabolites produced by molds. Mycotoxicoses are examples of poisoning as a result of exposure (mostly dietary but sometimes respiratory or even dermal) to mycotoxins. They may be acute or chronic and generally affect more people in developing countries, where they can worsen the effects of vitamin deficiency and malnutrition [191]. Mycotoxins are absorbed in the upper parts of the GIT, but to a greatly different degree that varies between more than 80% (aflatoxins) to less than 10% (fumonisins) [192]. Many mycotoxins can permeate the skin, although not, it seems, in sufficient doses to cause serious health problems [193].

The number of currently known mycotoxins varies between sources, but it is probably between 400 and 500. They are extremely diverse chemically but, unlike many bacterial toxins, are not proteins. Most of them are produced by relatively few genera of fungi. Those most hazardous to human health are briefly described below in Table 5.

Aflatoxins are the most important group of mycotoxins concerning human health. Over a dozen different aflatoxins are known, the four major ones being B_1_, B_2_, G_1_, and G_2_, classified according to their green or blue fluorescence under UV light. *Aspergillus flavus* and a few others from the same genus are the best-known producers of aflatoxins. Aflatoxin B_1_ (AFB_1_) is usually the major aflatoxin produced by toxigenic strains. It is one of the most potent carcinogens yet discovered, especially associated with liver cancer in chronic aflatoxicosis. Acute poisoning with aflatoxin is rare but could be fatal [191]. The death of 13 children in northwestern Malaysia in 1988 from acute hepatic encephalopathy and of at least 125 people (from 317 cases) in Kenya in 2004 were traced to Chinese noodles and homegrown maize, respectively, contaminated with aflatoxins [195].

Ochratoxin A (OTA) is comparable in importance to the aflatoxins, usually produced by many *Aspergillus* spp. and at least two *Penicillium* spp. (*P. nordicum* and *P. verrucosum*). It is often found in infected barley, oats, rye, wheat, coffee beans, and other plants of commercial value. OTA is a potent nephrotoxin to all animal species, associated with porcine nephropathy in Denmark and endemic nephropathy in Balkan countries such as Bulgaria, Romania, and the former Yugoslavia [191]. OTA half-life in humans can be as long as 35 days, considerably longer than in mice, pigs, or rats. Acute renal failure in humans has been associated with long-term exposure to ochratoxins in an agricultural setting [195,196].

Zearalenone (ZEA) is produced by *Fusarium* spp. and is most often found in cereals (especially maize). It is a structural analog of 17β-estradiol. Widely studied in various animal models (pigs, ruminants, mice), ZEA is best-known for its strong estrogenic and anabolic effects, to a lesser extent, hemato- and hepatotoxic effects. In pregnant women, long-term consumption of foods contaminated with ZEA presumably leads to reduced fetal weight and milk production; even changes in uterine tissue morphology have been suggested [195,197].

Among fumonisins, fumonisin B_1_ is the most prominent and the most toxic member of this group produced by *Fusarium* spp., which grows as corn endophytes. The toxins inhibit the synthesis of sphingolipids and cause various diseases depending on the species and the dose. In humans, fumonisins are strongly associated with esophageal cancer, especially in South Africa, China, and northeast Italy. Together with deoxynivalenol, fumonisins have also been implicated in the suppression of the immune response, for instance, significantly decreased levels of IL-8, IL-1β, IL-6, and macrophage inflammatory protein (MIP)-1β in piglets [200].

Trichothecenes are a large family divided into four groups. Groups A and B, produced by *Fusarium* spp., include all trichothecenes of major importance, namely T-2, HT-2, and deoxynivalenol (DON); groups C and D include less important members such as crotocin, verrucarins, and others. DON (aka vomitoxin or food refusal factor) may cause nausea, vomiting, and diarrhea in farm animals if ingested in high doses. Trichothecenes are commonly found in various grains (corn, barley, rye, wheat) and are strongly associated with alimentary toxic aleukia (ATA), whose acute phase is characterized by necrosis of the oral cavity, bleeding from various organs (nose, mouth, vagina), and CNS disorders. It was common in 19th-century Russia and the former Soviet Union, for instance, in the Orenburg district during the Second World War, when a large number of people got sick from eating overwintered grain infected with *Fusarium* [191,195,198].

Patulin was first isolated in the 1940s from *Penicilium patulum* (later renamed *P. urticae* and *P. griseofulvum*), tested as an antibiotic in the 1950s, and classified as a mycotoxin in the 1960s. Nowadays, patulin contamination most often comes from *P. expansum*, the blue mold that causes the soft rot of apples, pears, cherries, and other fruits [195].

Citrinin was originally isolated from *P. citrinum*, later also from a dozen of other Penicillium spp. (including *P. camemberti* of cheese fame) and several *Aspergillus* (such as *A. oryzae* used to make sake, miso, and soy sauce). Citrinin is a nephrotoxin in all species tested, although toxic doses vary greatly. It is found in many kinds of cereal as well as in some naturally fermented sausages in Italy [191].

Ergot alkaloids are a toxic cocktail found in the sclerotia of *Claviceps* spp., common pathogens on various grasses and grains, and are known, as well as the ergotism, from antiquity. It was a scourge in Europe during the Middle Ages when its two forms, gangrenous and convulsive, were responsible for high-mortality outbreaks; some 20,000 people were believed to have died from the disease only in the Aquitaine region in 944–945 AD. Though rare in humans nowadays, ergotism remains a major veterinarian problem [195].

### 4.2. LAB Detoxification of Mycotoxins

Mycotoxins are highly resistant to harsh conditions, including high temperatures during cooking, which makes them particularly difficult to be eliminated from contaminated foods. Crops may be contaminated with mycotoxins in the field, but this usually happens during prolonged and poor storage. No actual or precise figures about the worldwide loss due to fungal growth and mycotoxin production are available, but 25% of feed and food annually sounds like a reasonable estimation, which makes mycotoxins almost as much an economic threat as they are a health hazard [201].

Synthetic antifungal preservatives such as benzoate, sorbate, and propionate have been implicated in health issues ranging from irritability and inattentiveness to cancer and damage to the nervous system. LAB are safer and more desirable antifungal preservatives [202,203]. The antifungal properties of LAB are two major and essentially different types: (i) inhibition of fungal growth and (ii) neutralization of mycotoxins. Several studies have reported a broad spectrum of antifungal activity by many *Lactobacillus* spp. due to various organic acids production [204,205]. According to Lavermicocca et al. [206], PLA and OH-PLA synthesized by *Lp. plantarum* have anti-mold activity against *Aspergillus*, *Penicillium*, *Eurotium*, *Endomyces*, and *Monilia* as the minimum fungistatic concentration of PLA is 7.5 mg/mL, and the minimum fungicidal concentration is 10 mg/mL. These results are similar to the effect of caproic acid produced by *Fr. sanfranciscensis* CB1 against spoilage of bread by *Fusarium*, *Monilia*, *Penicillium*, and *Aspergillus*. PLA, ILA, and OH-PLA produced by *Lp. plantarum* and *Lentilactobacillus buchneri* have been shown to inhibit the growth of *Penicillium nordicum* and the synthesis of mycotoxins [207].

Besides antagonists to fungi, LAB are an antidote to mycotoxins. However, the exact mechanisms of this action remain elusive. The most studied is the adsorption of mycotoxins on the cell surface of LAB, where a complex network of teichoic and lipoteichoic acids, S-layer proteins, and exopolysaccharides plays a vital role in the process. The peptidoglycan layer has also been implicated. However, the binding capacity is highly variable: species- and strain-specific, greatly affected by pH and temperature, and mostly reversible [204,208]. Other mechanisms, such as the degradation of mycotoxins or their conversion to less toxic metabolites, are still waiting for proper experimental support [201]. Some remarkable feats of detoxification are summarized in Table 6.

#### 4.2.1. LAB against Aflatoxin B_1_ (AFB_1_)

AFB_1_ in cereals and cereal‐based products, to a lesser extent its less toxic but still dangerous metabolite AFM_1_ in milk and fermented milk products, remains a major global health problem for mycotoxins. Binding on the cell wall is the major mechanism by which LAB neutralize aflatoxins. Of 20 strains of LAB and bifidobacteria tested by Peltonen et al. [209], the most efficient were *L. amylovorus* CSCC 5160 and CSCC 5197, and *Lc. rhamnosus* Lc1/3. They were able to bind more than 50% of AFB_1_ from solution (5 μg/mL), 59.7, 57.8 and 54.6%, respectively, within 24 h. However, the binding was reversible. Upon incubation in toxin-free solution, various amounts of AFB_1_, 48.6, 30.7, and 26.5% for CSCC 5160, CSCC 5197, and Lc1/3, respectively, were dissociated from the bacteria and released back into the medium. Of the three *Lactococcus* strains studied, the most efficient proved to be *Lactococcus lactis* ssp. *cremoris* ARH74 with 41.1% binding of AFB_1_ [209]. Hence, favorable binding kinetics are necessary but, in itself, not a sufficient condition for a successful anti-mycotoxin probiotic. The cell count and the type of medium are important factors that may have a decisive influence. *Lc. rhamnosus* LBGG and LC-705 achieved 80% removal of AFB_1_ (5 μg/mL) from liquid media. The process was very rapid, reaching maximum in the very beginning and maintaining similar values for the next 72 h. Strains of *L. gasseri*, *L. acidophilus*, and *Lc. casei* were also tested, but their binding capacity was significantly lower and less consistent in time. Notably, however, even LBGG and LC-705 required very high cell densities, approximately 2 × 10^9^ CFU/mL, for effective detoxification. This makes the strains somewhat unsuitable as toxin-protecting food additives [210].

Of 11 LAB strains isolated from kefir, *L. kefiri* KFLM3 proved to be the most potent in eliminating AFB_1_ (1 μg/mL). Toxin binding, the assumed mechanism, was reversible and very much dependent on the pH and the medium. The AFB_1_ binding capacity of *L. kefiri* KFLM3 improved from 0% in MRS to 80% in milk. The bacteria/mycotoxin complex was found to be more stable at pH 7–8 and more prone to dissociate at pH 3: 12 and 37%, respectively, of the bound AFB_1_ were recovered [213]. LAB strains isolated from Brazilian artisanal cheeses were able to reduce the AFB_1_ levels much more effectively in phosphate buffer (>80% for some) compared to milk (>50% for all). The binding was time- and pH-dependent as well and, on the whole, much more effective close to neutral levels (6.5) than in a highly acidic environment (pH 3.0) and slightly better for 5 than for 15 min [215].

While the probiotic design is difficult under such conditions, it has been attempted in specific settings. *Lc. paracasei* LOCK 0920, *Lev. brevis* LOCK 9044 and *Lp. plantarum* LOCK 0945 achieved dose-dependent detoxification of broiler feed: 55% when contaminated with a low concentration of AFB_1_ (1 mg/kg) and 39% when contaminated with a high concentration of AFB_1_ (5 mg/kg). These results were obtained after 6 h of fermentation and remained stable 12 and 24 h after adding the strains, which the authors finally evaluated as a promising probiotic supplement for broiler feed [211]. An innovative study of ten LAB strains isolated from Brazilian artisanal cheeses, most notably *Levilactobacillus* spp. 3QB398, *Lp. plantarum* 3QB350 and *Lev. brevis* 2QB422 were shown to inhibit the production of aflatoxins B_1_, B_2_, G_1_, and G_2_ by *A. parasiticus*. The authors found that the time of inoculation with the LAB strains, simultaneously with the fungus or 24/48 h later, was critical for inhibition of the AFB_1_ production. Curiously enough, on the whole, these LAB strains appeared to be least effective against the most important aflatoxin, AFB_1_. Nevertheless, there were some notable exceptions. Three *Lp. plantarum* strains, 1QB147, 1QB314, and 3QB350, were able to reduce AFB_1_ production by more than 50%. *Levilactobacillus* spp. 2QB383 was the only strain with something like 100% effectiveness: even when it was inoculated 48 h after the fungus, no detectable levels of AFB_1_ were observed [215].

At least two different mechanisms, the involvement of bacteriocins and transcriptional inhibition of aflatoxin production, have been proposed based on some experimental evidence. Mixed culture of *Lp. plantarum* and *Lactococcus lactis* achieved an 81% reduction of AFB_1_ (0.05 μg/mL) in MRS broth after only 6 h of cultivation, and that level remained stable for another 24 h. This was considerably better than both species separately (46% and 27%, respectively) or common food preservatives such as benzoic and propionic acids (39% and 6%, respectively). The authors speculated that bacteriocins are largely responsible for the effect because they obtained their best detoxification values (90%) with a crude protein extract filtered through a 1000-Da dialysis membrane [212]. One of the few studies to propose a more sophisticated mechanism of LAB action against mycotoxins was published by Gomaa et al. [214]. Of 38 *Lactobacillus* species isolated from dairy products, *Lev. brevis* NM101-1 and *Lp. paracasei* ABRIINW.F58 were selected for their conventional antifungal activity (i.e., growth inhibition). This was found to be due to an antifungal compound of protein nature which remained active within a large range of temperatures and pH but lost its inhibitory effect upon treatment with proteases. Most interestingly, these antifungal compounds caused significant inhibition on the transcriptional level of the *omt-A* gene, which encodes a key enzyme in the biosynthesis of AFB_1_. The effect was species-dependent, more pronounced with the compounds from *Lev. brevis*, which reached 80 and 64.5% inhibition of *A. flavus* and *A. parasiticus*, respectively. The antifungal compounds from *Lc. paracasei* were somewhat weaker but still reached 70 and 52% inhibition, respectively, of the *omt-A* gene in the same two *Aspergillus* spp. [214].

In regard to LAB and AFB_1_, it may be concluded that the suitable strains for effective detoxification are relatively few and need rigorous testing before they are approved as probiotics.

#### 4.2.2. LAB against Ochratoxin A (OTA)

Adsorption on the cell wall of LAB appears to be the most predominant mechanism of detoxification of OTA [196]. Yogurt bacteria are capable of remarkable reduction of OTA content in milk. *Str. thermophilus* T4 and *L. bulgaricus* LB-51 achieved complete elimination of 0.5 mg/L OTA after 18 h of incubation; and 36 and 26% drop of OTA with concentrations of 1.0 and 1.5 mg/L, respectively. The strains were less effective separately, with 79% and 62% OTA removal for *Str. thermophilus* and *L. bulgaricus*, respectively. The authors reported a change in morphology in the lactobacilli (longer rods, thinner cell walls) at high OTA concentrations [216]. Another strain of *L. bulgaricus*, 259/2 was able to reduce OTA (with a concentration of 50 ppb) by 94% after 48 h incubation in MRS medium. However, other studies showed great variability in OTA binding by *L. bulgaricus* (6 to 34%), which implies great strain specificity [231]. Two strains of *L. acidophilus* (1A and 4A) were also able to reduce OTA by 46.5–32.7% [217]; and *L. helveticus*—by between 67.1 and 71.9% [217,231]. Notably, different authors used various OTA concentrations (50–1000 ppb) and media; therefore, the data comparison was difficult. For example, *L. kefiri* KFLM3 decreased OTA (1 μg/mL) by 81% in milk but only by 15% in MRS [213].

A very impressive degree of detoxification of OTA has been achieved by LAB in Douro wines. *P. parvulus* strains achieved 89–98% degradation of OTA (1 μg/mL) in MRS medium after 5 days of incubation at 30 °C with 10^3^ CFU/mL. *P. parvulus* UTAD 473 reached 100% degradation of OTA under these conditions; 16 other LAB strains (mostly *Lp. plantarum* and *Oenococcus oeni*) also decreased OTA by 10–20%. The rate of the process was dependent on the inoculum size (almost five times faster with 10^9^ CFU/mL) and the incubation temperature (~30% slower at 37 °C). The presence of ochratoxin α (a degradation product of OTA) was confirmed by LC-MS/MS, which suggested peptidase activity displayed by the strain. The study of OTA adsorption on *P. parvulus* cells was only 1.3%, thus suggesting that the main mechanism of detoxication by this strain is OTA degradation [220].

Another study of OTA degradation compared 27 commercial LAB strains cultivated in MRS contaminated with 0.6 μg/mL OTA for 24 h at 37 °C. The authors concluded that among the six strains that showed 97–99% total reduction of OTA at pH 6.5, hydrolysis was by far the predominant mechanism; only 2–4% were due to adsorption. Curiously, the hydrolysis was less effective in a more acidic medium (pH 3.5). Degradation products ochratoxin α and phenylalanine were confirmed by mass spectrometry [221].

Interestingly, a study of OTA reduction by *L. bulgaricus* also tested the ability of these lactobacilli to neutralize several different trichothecenes, such as nivalenol (1 ppm), deoxynivalenol (1 ppm), diacetoxyscirpenol (500 ppb), and T2 toxin (500 ppb), but no effect was observed [217]. The same lack of correlation between detoxifying capacities was demonstrated for OTA and patulin by *Lactobacillus* and *Bifidobacterium* [219]. On the whole, the efficiency of LAB as OTA scavengers is considerable and reinforces their role as probiotics with anti-mycotoxin action. However, as in the case of AFB_1_ detoxification, strains must be selected with great care regarding their capacity to neutralize OTA and their optimal conditions.

#### 4.2.3. LAB against Patulin

In recent years, perhaps because of its easy availability on moldy fruits, patulin has attracted some notable attention in the field of LAB detoxification. An intriguing study with heat-inactivated LAB used methods such as Fourier Transform Infrared Spectroscopy (FTIR), Zeta Potential, and Contact Angle to confirm the importance of physical and chemical parameters such as specific surface area, cell wall volume, and N/C ratio for the binding capacity of patulin. Since CO-, OH-, and NH- were the main functional groups involved, probably polysaccharides and/or proteins are the crucial binding molecules. Among the studied LAB, *Lev. brevis* 20,023 was found to have the highest specific surface area, greatest cell wall volume, and, expectedly, highest capacity (65.02%) to adsorb patulin (4 mg/L) from aqueous solution [222]. *Lp. plantarum* ATCC 8014 achieved 96% patulin removal from apple juice during 6 weeks of cold storage after the juice was purposefully contaminated with 100 μg/L of the toxin. However, very high cell density was required (3.6 × 10^11^ CFU/mL), as well as the addition of prebiotic fructooligosaccharide (2.3%), ascorbic acid (213 mg/L), and citric acid (1.4 g/L). SDS-PAGE was used to confirm that S-layer proteins were involved in the adsorption of patulin. The electrophoresis showed a sharp decline in the amount of a 50-kDa fraction on the first day of incubation, which is in agreement with the kinetics of patulin decrease: almost 70% on the first day, a much slower but steady decrease until the 42nd day [223]. A recent study used LAB from Tibetan kefir grains for the detoxification of apple juice and went into some detail about the adsorption mechanism. FTIR was used to establish the most important functional groups, and while the result (C–O, OH, C–H, N–O) was somewhat different from the study mentioned above [222], the authors reached the same, admittedly rather general, conclusion: Proteins and polysaccharides on the cell surface must be responsible for the patulin adsorption. Of the five strains tested, *L. kefiranofaciens* JKSP109 was the finest patulin scavenger—93% at 100 μg/L but only 56% at 200 μg/L. The adsorption capacity was found to depend on pH and the °Brix, in which the higher, the better in both cases [224].

#### 4.2.4. LAB against Deoxynivalenol (DON), Fumonisins, and Zearalenone (ZEA)

DON has been a somewhat unpopular research subject in the last few decades, which is surprising considering its prevalence in cereal crops. According to some studies, 65% of the maize kernels harvested in France from 2004–2006 were contaminated with DON and fumonisins; another study of corn samples from several European countries found that 52 of 67 contaminated samples (78%) contained DON and while only two of them exceeded the EU recommended values (8 mg/kg in grain and grain products), six others exceeded 1 mg/kg; concentrations from 100 to 1000 μg/kg appeared to be quite common in Europe [192,232]. A couple of recent studies have dealt with LAB as DON detractors in a somewhat illuminating way.

Altogether 16 LAB strains, eight commercially available in probiotic formulae (e.g., Lyofast LPRA, Yo-flex YC-180), and eight isolated from cereals and kefir (mostly *Lp. plantarum*), were tested for anti-fungal activity and DON reduction. Six of them significantly inhibited the growth (agar halos bigger than 30 mm in diameter) of *Fusarium graminearum* JAPAR 2218, a confirmed DON producer and an economic scourge for grain crops worldwide. DON reduction studies were conducted with 1.5 μg/mL toxin in MRS for 4 h in a volume of 2 mL with average cell densities of 10^10^ CFU/mL and three types of bacteria, viable and heat-inactivated. In all cases, the sterilized cells showed a better ability to reduce DON, usually 20–30% higher than that of the viable cells. However, the best in DON detoxication *Lp. plantarum* GT III (67% decrease), was not the most potent fungicide (27 mm halo) [225]. *Lc. paracasei* LHZ-1 isolated from yogurt achieved a 40.7% reduction of DON (50 μg/mL) by the cell wall fraction in PBS for 24 h at 37 °C. In contrast, only 10.5% and 8.9% were reduced by culture supernatant or cellular lysate, respectively. Laser scanning confocal microscopy was used to elucidate further the mechanism of DON detoxification. DON was labeled with AMCA-X SE to produce blue fluorescence and thus obtained visual evidence that DON does form complexes with the bacterial cell wall. As in the aforementioned study [225], pasteurized and sterilized cells removed DON more efficiently than viable cells, only in this case, the increase was only 5–6% at most.

After DON, fumonisins are the next most prominent contaminants of food and feed [232]. It was found that LAB starter culture (*Lactococcus lactis*, *L. delbrueckii*) added to a maize meal could reduce the levels of fumonisin B_1_ (2 μg/g meal) by almost 75% for 4 days. This fermented meal was comparatively less toxic to SNO human esophageal carcinoma cell line, but the difference was not significant. The authors perceptively note that the reduction of the toxin level may not necessarily result in reduced toxicity because the LAB fermentation does not alter the bioavailability of the toxin. Chronic complications from trace amounts of the toxin remain a potential problem [227]. Another study provided some insight into the exact components of the LAB cell wall that bind fumonisin B_1_ and B_2_ (FB_1_, FB_2_). The importance of peptidoglycan (PG) was confirmed in two different ways. Mutants with defective PG layer displayed decreased toxin binding, which affected only FB_2_, and only with 20–25%. Purified PG bound fumonisins (5 μg/mL each) in a similar, but somewhat lower, degree to LAB (20% for FB_1_, 60% for FB_2_, both at 2 mg/mL PG). Mutants with a defective synthesis of lipoteichoic acids showed negligible difference (5–10%) compared to the wild type, indicating that this component of the cell wall is unimportant as far as fumonisin binding is concerned. The tricarballylic acid chains of the fumonisins were confirmed to be essential for the toxin-binding, which decreased when the chains were hydrolyzed. The authors also claimed that treatment with lipases and proteases had no effect on the toxin binding, and neither did the use of mutants lacking exopolysaccharides [228].

Zearalenone (ZEA) has been reported in foods and body fluids (animal as well as human) with an alarming frequency [197]. As in the cases of AFB_1_ and OTA, a great deal of work has been done on LAB detoxification of ZEA, but the molecular mechanisms remain elusive. One promising probiotic of the future against ZEA is *Lp. plantarum* A1, a strain with a potent and rapid ability to bind ZEA (20 μg/mL). The process was partially reversible, dropping from immediate 99% to 77% after 72 h cultivation in MRS broth, but the relatively small inoculum (10^8^ CFU/mL) was a point in the strain’s favor [229]. Similar kinetics were obtained with *Lactococcus lactis* isolated from milk products and 130 μg/mL ZEA, although in this case, the process appeared to be virtually irreversible [230]. LAB starter culture (*Lactococcus lactis*, *L. delbrueckii*) added to a maize meal reduced the levels of ZEA (2 μg/g meal) by 68% for 4 days; as in the case of FB_1_, the decreased toxicity on the SNO cell line was not significant [227]. *L. kefiri* KFLM3 achieved a 100% decrease of ZEA (1 μg/mL) in milk, but only 60% in MRS, yet another reminder of the importance of the medium [213]. *Lp. plantarum* 3QB361, isolated from Brazilian cheese and inactivated in phosphate buffer, managed to reduce ZEA (2 μg/mL) with 70–80% at pH 6.5, but five other strains (from ten tested) hardly managed 20–40%—another timely reminder, this time of species- and strain-specificity [215].

## 5. Lactic Acid Bacteria for Reducing Pesticide Levels in Food

The toxic effects of various pesticides in humans include neurotoxicity, skin irritation, carcinogenesis, and endocrine disruption [15]. Among the symptoms are abdominal pain, nausea, vomiting, diarrhea, headache, lethargy, tremor, muscle spasm, coma, kidney insufficiency, upper airway and mucous membrane irritation, tachycardia, weakness, acidosis, hypotension, ataxia, hypertonia, etc. [4]. For instance, the mechanism of action of organophosphate pesticides is to inhibit acetylcholinesterase, which leads to an impaired connection between acetylcholine and its receptor in nerve and muscle cells. The toxicology research also shows that pesticide exposure induces oxidative stress and DNA, protein, and lipid damage, followed by adverse health and psychological effects [16,233].

There are several types of pesticides according to their chemical structure: organochlorine, organophosphorus, neonicotinoid, benzimidazoles, carbamates, and synthetic pyrethroids. Organochlorine pesticides, also known as “contact” insecticides, can be accumulated in fatty tissues and milk. They are highly persistent in the environment, and for this reason, their application in most countries is prohibited. The oldest and best-known organochlorine is the insecticide DDT (1, 1, 1-trichloro-2,2- bis (4 -chlorophenyl) ethane); it is a usual contaminant of hen eggs and milk products. Common organophosphate pesticides are chlorethoxyfos, chlorpyrifos, and diazinon, esters of ortho-, thio-, and pyro-phosphoric acids. Organophosphate pesticides act as acaricides but primarily as insecticides. They are highly toxic to bees, wildlife, and humans. Urea Pesticides are another class of herbicides; they are inhibitors of photosynthesis in plants. The most commonly applied among them are isoproturon, chlortoluron, and fluometuron. Other herbicides are dinitroaniline pesticides (trifluralin; pendimethalin; oryzalin; prodiamine; ethalfluralin; benfluralin). The organophosphate pesticides can be found in foods such as milk and yogurt, wheat flour, cabbage, eggplants, cucumbers, maize, and tomatoes. Carbamates are selective herbicides, insecticides, acaricides, nematicides, molluscicides, or fungicides in fruits and vegetables. The most widespread carbamate pesticide, aldicarb, found in high concentrations in watermelons, caused food poisoning that affected more than 2000 people in the USA in 1985 [234]. Quaternary ammonium salts (paraquat; diquat; chlormequat) are the most toxic of all insecticides or herbicides. Nearly 25% of the global market for insecticides is occupied by the class of neuro-active insecticides neonicotinoids (chemically similar to nicotine).

Many LAB of *Lactobacillus* and *Leuconostoc* genera can metabolize a broad spectrum of synthetic insecticides and use them as carbon and energy source. The mode of action is through their esterase and phosphatase enzymes [13]. DDT degradation (1 ppm in milk and cheese) by *Str. thermophilus* and *L. bulgaricus* was shown by Abou-Arab two decades ago [235]; however, the drop was only 10.8–11.8%. Later, *La. sakei* pro7 isolated from soil reached 95.1% biodegradation of DDT with a concentration of 20 ppm [236]. The following LAB able to convert chlorpyrifos were isolated from kimchi: *Leuc. mesenteroides* WCP907, *Lp. plantarum* WCP931, *La. sakei* WCP904, and *Lev. brevis* WCP902 [237]. The last strain consumed 83.3% of 30 mg/l of the pesticide in 3 days and completely assimilated it after 9 days. In search of the molecular mechanisms of degradation, the responsible *opdB* gene was determined, cloned, and the relevant enzyme OpdB (274 amino acids) was purified. It contains the “Gly-X-Ser-X-Gly” motif typical for bacterial organophosphorus hydrolases and is a member of the esterase family [238]. Kimchi LAB strains are also known to degrade coumaphos, diazinon, methylparathion, and parathion [237]. Recently, Maden and Kumral [239] investigated the degradation of insecticides in sauerkraut samples with or without the presence of lactic acid bacteria during fermentation. *Lp. plantarum* 112 (previously isolated from olive brines, 10^9^ CFU/mL) contributed for malathion (2 mg/kg) and chlorpyrifos-methyl (4 mg/kg) degradation. However, the decrease of λ-cyhalothrin was low. The same team [240] tested *Lp. plantarum* strains for pesticide removal in the course of black olive fermentation. At the end of fermentation (after 60 days), 61% of deltamethrin, 68% of dimethoate, and 50% of imidacloprid were removed by *Lp. plantarum* 123. Significant success was achieved in the detoxification from synthetic pyrethroids. Dorđević et al. [241] underlined the role of LAB in bifenthrin removal from wheat flour; then *Lp. pentosus* 3–27 was applied for the successful elimination of beta-cypermethrin from silage [242]. The strain degraded 96% of the pesticide with a concentration of 50 mg/L. LAB species and strains capable of removing pesticides from foods are shown in Table 7.

As summarized by Mohammadi et al. [249], the most common mechanism of pesticide elimination by LAB is the enzymatic hydrolysis by carboxylesterases, organophosphate hydrolases, phosphotriesterases, and phosphatases. That is why LAB are potent detoxifiers of food from organochlorine, organophosphorus, and pyrethroids, but there is no evidence that they can degrade carbamate pesticides. However, some species of lactobacilli, such as the sourdough isolate *Fru. sanfranciscensis* DSM 20451^T^ are highly resistant to the carbamate paraquat [250], while others (such as the probiotic *Li. fermentum*) have been used successfully to alleviate oxidative stress in piglets caused by diquat [251].

## 6. LAB against Heavy Metals Intoxication

Foodstuff can also be contaminated with other toxic and non-degradable elements such as heavy metals. Heavy metals are defined as metallic elements with a density above 5 g/L [252]. Most metals toxic to human health are considered cadmium (Cd), lead (Pb), mercury (Hg), arsenic (As), and chromium (Cr) [253,254]. However, even in low concentrations, many physiologically essential for the human body heavy metals such as iron (Fe), zinc (Zn), copper (Cu), cobalt (Co), manganese (Mn), etc., can also be hazardous [254]. Sources of heavy metal pollution are several industries [252], pesticide and veterinary drug residues [255], packaging materials [256], technological incidents, and many others, which contaminate foodstuff and drinking water directly or by distribution in the environment and slow accumulation in food chains through polluted agricultural soils or intoxicated aquatic animals [257]. In case of prolonged ingestion, heavy metals accumulate in the human body, adversely affecting the nervous, cardiovascular, and reproductive systems, causing renal and lung diseases, hepatic damage, skin problems, and bone demineralization [258,259,260,261,262,263]. Moreover, most heavy metals are defined as carcinogenic (e.g., As, Cd, Cr) or possibly carcinogenic (e.g., Pb) to humans [264]. In oral intoxication, the gastrointestinal tract is the first organ where metals are absorbed [263], but once in the bloodstream, they accumulate mainly in the kidney and liver [265,266]. In addition, long-term exposure to both Cd and Pb disrupts calcium homeostasis and causes mitochondrial damage [267] and oxidative stress leading to lipid peroxidation [268] and DNA fragmentation [269]. On the other hand, arsenic, as a proven genotoxic agent, can cause skin, lung, and kidney cancer in case of prolonged intoxication [270].

Current methods for metal detoxification are divided into physical, chemical, and biological [271]. The most employed techniques for metal removal from contaminated industrial areas, waters, and the environment are chemical precipitation, ion exchange, membrane filtration, and solvent extraction [272,273,274]. Detoxification of heavy metals in vivo has been achieved with various chelating agents such as dimercaptosuccinic acid (DMSA) and ethylenediaminetetraacetic acid (EDTA), which promote the excretion of heavy metals [252]. However, the described chemical methods for both in vitro and in vivo metal detoxification have serious drawbacks limiting their application. The chemical methods designed to detoxify the environment are extremely expensive or ineffective and generate additional toxic waste [275,276,277]. On the other hand, detoxification by chelators is effective but not suitable for prolonged treatment [278] due to safety concerns [279,280].

Biological methods based on biosorption of metals or metal-containing compounds by plants [281], algae [277,282], bacteria [283], and fungi [284] are promising options due to their high efficiency and specificity, lack of side effects, and low investment cost [285]. The biosorbents have the capacity to decrease heavy metal concentration from ppm to ppb levels in aqueous solutions [277]. Although the process of metal biosorption is initially well studied in other organisms (e.g., *Aspergillus* spp., *Penicillium* spp., *Bacillus* spp., *Saccharomyces* cerevisiae, etc.), the use of LAB as a biosorbent is of particular interest, due to their GRAS status and probiotic nature. Moreover, LAB could be easily added to the diet in an attempt to alleviate heavy metal intoxication in the human body.

As with other organisms, the capacity for metal removal by LAB is strain-specific and depends on many factors such as cell surface content, protein production, pH of the environment, temperature, type of metal element, and both cell and metal concentrations [254,286]. The overall process consists of two distinct mechanisms: binding of metals to the bacterial cell wall by electrostatic interaction (biosorption) and passage of metal ions through the cell membrane and accumulation inside the cell (bioaccumulation) [256]. The former mechanism is fast and metabolically independent; the latter is slow, requires metabolic activity, and takes place only when biosorption has reached its limit [287].

As Gram-positive bacteria, LAB possess on their cell wall surface a thick layer of peptidoglycan, teichoic acids, and S-layer proteins, which play a key role in the ability to bind and sequester metals by ion-exchange reactions [288]. LAB have a negatively charged surface and are suitable for binding cations such as Hg^2+^, Cd^2+^, Pb^2+^, etc. [289]. Moreover, LAB strains, which produce exopolysaccharides, have additional negatively charged groups on their surface (carboxyl, hydroxyl, phosphate) and an additional number of ligands for binding metal cations [290]. When the negatively charged group on LAB cell wall surfaces, such as carboxyl, phosphoryl, or carboxylate, are neutralized, the binding properties of the bacterium sharply decrease [291,292,293]. Environmental pH is another factor that strongly affects biosorption in LAB. Adsorption of Cd and Pb is very low at pH ≤ 3.0 and gradually increase above 3.0 to reach its maximum between pH 4.0 and pH 6.0 [252]. For example, the best rates of metal removal by *Li. fermentum* ME3, *Lc. rhamnosus* GG, and *L. acidophilus* X37 were observed at pH 6.0, for *L. bulgaricus*—at pH 5.0 [271]. Likewise, the production of specific proteins appears to be of vital importance for LAB biosorption. Kinoshita et al. [286] identified ~14 kDa mercury-binding protein from the cell surface of *Weissela viridescens* MYU 205. This protein contains the “CXXC” motif (as “X” is any amino acid), which is a well-known heavy metal-binding motif contained in various proteins with the confirmed binding ability of Cd^2+^, Co^2+^, Cu^2+^, and Zn^2+^ [294,295].

Since biosorption is not a metabolically connected mechanism, LAB can be used for metal removal both in viable and non-viable conditions. Several authors suggest that living cells have a higher binding capacity than dead ones [296,297,298]. Tian et al. [298] studied the binding capacities to cooper of 16 different LAB strains, and the results showed that all of them have a higher binding capacity as living cells. In another study, however, *Lp. plantarum* PTCC 1896 showed an increased biosorption of Cd^2+^ and decreased—of Pb^2+^ when the cells were heat-killed [299]. *W. viridescens* MY 205 showed decreased removal of Cd^2+^ and Pb^2+^ but increased—of Hg^2+^ after cells’ heat inactivation [300]. A probable explanation of these results is that the change in sorption capacity after inactivation might be dependent on both the strain and type of metal. Nevertheless, LAB could be applied successfully for heavy metal detoxification as a viable culture without losing their probiotic characteristics. 

For selection as suitable biosorbents, LAB strains are tested for their metal-resistant and metal-removal abilities. To assess the metal resistance of the strain is used the term minimum inhibitory concentration (MIC), which is the lowest metal concentration that completely inhibits the growth of the strain [301]. LAB strains displayed a wide spectrum of MIC values. Bhakta et al. [302] tested 26 LAB strains for Cd- and Pb-resistance and reported that the MIC values for Cd are in the range from 50 to >1000 mg/L for the different strains, while for Pb are >2000 mg/L for all strains tested. It is considered that strains with MIC values exceeding those of the control organism *E. coli* K-12 (e.g., MIC >100 mg/L for Cd and MIC > 1600 for Pb) are tolerant to the respective metal [303]. This indicates that, in general, LAB strains are relatively resistant to heavy metals.

The metal-removal abilities of LAB have been confirmed in many in vitro and in vivo studies. The biosorption capacity is strain-specific, and rarely is a strain a good sorbent of many different metals. According to Kinoshita et al. [286], LAB exhibited the following order of preferential sorption in regard to the most toxic metals: Hg^2+^ > Cd^2+^ > Pb^2+^ = As^3+^. However, the same authors concluded that Hg most strongly inhibits bacterial growth due to its higher toxicity. On the other hand, LAB strains possess notably high levels of resistance to Pb, which allows the sorption performance at higher metal concentrations [255,299,304,305]. Contrariwise, the biosorption of arsenic can be implemented only at comparably low initial metal concentrations [289,306]. In addition, other potentially hazardous metals, such as Cu, Fe, and Zn, can be adsorbed very successfully by LAB strains in vitro [307,308]. The most successful metal biosorptions in vitro by living LAB strains are listed in Table 8.

Over the last decade, many in vivo studies have revealed that LAB strains (especially *Lactobacillus* spp.) have a remarkable influence on the heavy metal intoxicated human body. Orally taken by fermented foods consumption, they can detoxify different organs and tissues [288]. Thus, the prolonged intake of yogurt with concentrated cell culture of *Lc. rhamnosus* GR-1 can prevent further increment of Hg and As blood levels of pregnant women subjected to chronic exposure [278]. In experiments with mice, the addition of *Lp. plantarum* CCFM8610 and *L. bulgaricus* CCFM8004 in soymilk have a protective effect against chronic Cd exposure [309]. Jama et al. [310] used a combination of *Lc. rhamnosus*, *L. acidophilus*, and *Bifidobacterim longum* against Cd-induced genotoxicity in rats and succeeded in reducing it by 20%. Likewise, different *Lp. plantarum* strains are successfully applied for Cd sequestration in mice intestines [311], reduction of Pb levels in mice blood and tissues [296], and reduction of Al and Cu levels in mice livers, brains, and kidneys [298,312]. The authors connected the LAB antioxidative properties with the complex action on the metal intoxicated body.

In addition to metal adsorption in tissues, LAB also alleviate oxidative stress [311], protect the intestinal barrier [313], prevent losses of essential metals [298,311], and finally, remove metals from the body by defecation [286].

**Table 8:** In vitro biosorption of heavy metals by living LAB and *Bifidobacterium* strains.

Heavy Metal	Biosorbent	Initial Metal Concentration (mg/L)	Metal Removal (%)	Metal Removal Capacity (mg/g Dry Biomass)	References
Hg	*Weissella viridescens* MY 205	1	79.6		[300]
Cd	*Propionibacterium freudenreichii shermanii* JS	50	49.1		[305]
	*L. acidophilus* ATCC 20552	50	65.5		[304]
	*Lc. rhamnosus*, *L. acidophilus, Bifidobacterium longum*	43 ^a,^*	48.0		[310]
	*Bifidobacterium longum* 46	10		54.7	[314]
	*Ent. faecium* EF031	10	97.5		[315]
	*Lp. plantarum* PTCC 1896	10	90.9	122.7 ^b,c^	[299]
	*Lp. plantarum* CCFM8610	5	77.0	3.85	[311]
	*Li. reuteri* Cd70-13	1	25.0		[302]
	*P. acidilactici* As105-7	1		0.13 ^d^	[306]
	*W. viridescens* MY 205	1	54.1		[286]
Pb	*Lp. plantarum* LAB-32	200	82.25	57.31 ^b^	[255]
	*Lp. plantarum* PTCC 1896	50	65.4	34.5 ^b,c^	[299]
	*L. acidophilus* ATCC 20552	50	72.6		[304]
	*Propionibacterium freudenreichii shermanii* JS	50	69.9		[305]
	*Li. reuteri* Pb71-1	6	59.0		[302]
	*P. acidilactici* As105-7	6		0.76 ^d^	[306]
As	*L. acidophilus*	1	60.0		[316]
	*L. acidophilus* ATCC 20552	0.5	49.8		[304]
	*Lc. casei* DSM20011	0.1	38.1	0.312 ^c^	[289]
	*P. acidilactici* As102-4	0.1		0.006 ^d^	[306]
Al	*Lp. plantarum* CCFM639	50	26.83		[312]
Cu	*Ent. faecium*	250		106.38 ^c^	[308]
	*Lentilactobacillus buchneri* DSM 20057	40		46.17 ^c^	[317]
	*Lev. brevis*	20		26.5 ^c^	[318]
Fe	*L. bulgaricus* Lb-12	100	99.3		[307]
	*Str. thermophilus* STM-7	100	100.0		[307]
Zn	*L. bulgaricus* Lb-12	100	90.2		[307]
	*Str. thermophilus* STM-7	100	92.8		[307]
	*Leuc. mesenteroides*	20		27.10 ^c^	[319]
	*W. viridescens* MY 205	1	20.0		[300]

* Designations: ^a^, estimated from 70 ppm CdCl_2_; ^b^, mg removed metal per gram wet biomass; ^c^, maximum removal capacity, calculated from Langmuir isotherm; ^d^, metal removal efficiency (mg removed metal per hour per g wet biomass).

LAB can be successfully used for the metal detoxification of foods and drinks. For example, treatment with *Lp. plantarum* CCFM8610 removed up to 82% of the Cd from nine types of fruit and vegetable juices [320].

In conclusion, LAB have a high potential as a biosorbent of heavy metals both from foodstuff and from the intoxicated human body. LAB can be easily applied as a biosorbent in the form of food additive, especially in fermented foods and drinks, providing a comprehensive reduction in damage from metal intoxication.

## 7. LAB in Detoxication of Food from Natural Antinutrients

Leaf vegetables, legumes, and cereals food contain antinutrients—natural compounds that interfere with the absorption of nutrients. They are toxic or are a platform for toxic compound synthesis during their degradation in the human body. Examples of antinutrients include phytic acid, cyanogenic glycosides, oxalates, and protease inhibitors. Other chemicals considered to be antinutrients are toxic only in certain cases of insufficiently processed foods (lectins). Some of them have a controversial role in the human body (for example, phenolic acids have an antioxidant effect) and, so far, cannot be attributed to the group of antinutrients [321]. Phytate is one of the most studied antinutrients, as it can chelate various nutrients and reduce their bioavailability. It causes mineral deficiencies because it inhibits the absorption of zinc and iron in human GIT [322]. LAB fermentation is a good approach to diminish the adverse effect of phytate-rich cereals such as pearl millet and maize, but also other cereals and pseudocereals. These foods are a source of LAB displaying phytase activity, for instance, *Lp. plantarum* and *Li. fermentum* isolated from the fermented teff meal *injera* and the pearl-millet fermented gruel *ben-saalga* [323]. According to Sharma et al. [324], both *L. amylovorus* and *Lp. plantarum* from sourdough show high phytase activity: 125–146 U/mL and 60–74.4 U/mL, respectively. Traditional Asian sourdoughs for *dosa* and *idli* (made of rice and black gram dhal) are subjected to natural lactic acid fermentation for at least 20 h for reduction of the phytates content [324]. Castro-Alba et al. [325] fermented quinoa, amaranth, and canihua with *Lp. plantarum* 299v, thus reducing phytate concentrations by 47–51%, 12–14%, and 25–27%, respectively. However, the presence of some phytates and tannins in food and tea may decrease the bioaccessibility of mercury and prevent heavy metals poisoning [258].

Cyanogenic glycosides are the substrate that releases the respiratory inhibitor hydrogen cyanide after hydrolysis in the human organism. HCN is in lethal dose if consumed in an amount higher than 3.5 mg per kg body weight. Plant foods contain about 25 cyanogenic glycosides, such as linamarin (cassava, white clover, flaxseed), dhurin (in all sorghum cereals), prunazine, and amygdalin (apples, apricots, plums, almonds, cherries). Amygdalin in apricot kernels reaches 17.5 mg/g. Apple seeds can contain up to 4 mg/g of this glucoside, and that is why commercial apple juice usually contains 0.1 mg/mL of amygdalin [326]. LAB converting cyanogenic glycosides are relatively rare: among 25 strains representing 23 species of LAB screened by Menon et al. [327], only *Lp. plantarum* and *Lp. paraplantarum* grew well and degraded amygdalin, similarly to Lei et al. [328]. Linustatin, neolinustatin, and linamarin found in linseed were destroyed by *L. acidophilus*, reaching a 66% reduction in the total amount of cyanogenic glycosides. *L. delbrueckii* starter cultures were used for cyanide detoxification of Tanzanian cassava meal *Mchuchume* by decreasing cyanogenic glycosides from 72.72 to 5.18 mg/kg [329]. In all these cases, as during the LAB fermentation of bamboo [330] or hemp sourdough made by the use of *Lp. plantarum*, *P. acidilactici*, and *Leuc. mesenteroides* starter [331], a significant decrease in the concentration of phytic acid, tannins, and saponins, was observed during fermentation.

Oxalates—salts of oxalic acid occur naturally in many plants. In addition to being consumed, oxalates are also obtained in the human body as waste from the breakdown of food. Various (otherwise useful foods) are high in oxalates: leafy greens and legumes. The danger of consuming many oxalates comes from their ability to bind calcium, thus increasing the risk of kidney stones in some people. Oxalates consumption is linked to pathologic conditions such as hyperoxaluria, urolithiasis, renal failure, cardiomyopathy, and cardiac conductance disorders [332]. Several LAB species can degrade oxalates in vitro and in vivo. *L. acidophilus* breaks down 11.8% of 10 mM ammonium oxalate, while *Str. thermophilus*—2.3% [333]. Other species reducing oxalate absorption in GIT are *Lev. brevis*, *Lc. casei*, *L. gasseri*, *L. salivarius*, *Li. fermentum*, *Weissella confusa*, and *W. cibaria* [334]. Azcarate-Peril et al. [335] showed that *frc* and *oxc* genes encoding functional oxalate-degrading enzymes were identified in *L. acidophilus* NCFM and *L. gasseri* AM63T. However, one of the strongest oxalates destroyers is *Ent. faecalis*, which is “oxalotroph” and uses oxalates as a sole carbon source [336]. Hokama et al. [337] found that the oxalate-degrading *Ent. faecalis* produces three unique proteins involved in the oxalate degradation. Murru et al. [333] reported that *Lp. rhamnosus* GG diminishes the oxalates content of food in vitro. Currently, a number of probiotics are developed to prevent calcium oxalate urolithiasis [338].

Other substances classified as antinutrients are amylase/trypsin inhibitors or ATI. They are small, cysteine-rich proteins involved in the wheat defense system against insects and fungi. They are classified as antinutrients as they cause non-celiac wheat sensitivity (NCWS), an immunological disorder that shares the symptoms of celiac disease and irritable bowel syndrome [339]. LAB can hydrolyze ATI, as shown in vivo in mice [340]. Strains that showed the highest ATI-degrading activity are *Ligilactobacillus salivarius* H32.1, *Li. mucosae* D5a1, and *Lc. rhamnosus* LE3, as well as the sourdough, isolates *Fru. sanfranciscensis*, *Li. reuteri*, and *La. sakei* [341].

## 8. Conclusions

The 21st century is associated with food shortage, global warming, and increasing pollution of waters and soils. For instance, due to climate change, the aflatoxins that were common in hot and humid regions until now are expected to increase their deadly presence as major contaminants in European foods. The contemporary hopes of the food industry are in environmentally friendly approaches to food detoxification.

Although the action of LAB species as detoxifiers is usually limited to a specific toxicological agent, there are strains capable of significant and complex reduction in the amount of several toxic ingredients in food. Considering the wide spectrum of toxic food substances, LAB are most effective against mycotoxins and bacterial toxigenic producers. LAB’s major mechanisms are neutralizing the toxins, in one way or another, by metabolic degradation or biosorption, for example, and/or inhibiting the growth of the producers themselves. LAB are capable of enzymatic hydrolysis of several types of pesticides. The data in this direction are impressive and promising, and we may express certain hope for even better results in the future. The case of LAB against various antinutrients is likewise hopeful, although to a lesser degree. Concerning the detoxification of heavy metals, LAB act on the one hand preventively, purifying the soil and water used for food production, and on the other hand, as a means of combating the already existing poisoning of the human body. Here they can, at best, act as a probiotic remedy for more effective excretion of heavy metals from the body.

It should be noted that many toxins enter the food simultaneously and may act in combination, for example, mycotoxins and pesticides, bacterial toxins and antinutrients, mycotoxins and antinutrients, etc. In this case, LAB prove to be indispensable. During lactic acid fermentation, probiotic LAB strains achieve both toxins removal and the food’s nutritional value increase, especially with regard to foods of plant and cereal origin. In the future, the search for and application of new probiotic LAB strains for potential detoxification of mixed toxic agents is very promising and may be expected to become even more important than it is at present. The combination of different LAB strains with various detoxifying capabilities could serve as starter cultures for the production of safer and healthier functional foods.

## Figures and Tables

**Table 1:** Established mechanisms of antibacterial activity of lactic acid bacteria (LAB) against toxigenic *E. coli* strains.

*E. coli* Strain	LAB Species, Strain	Source/Model System	Agent/Bioactive Molecule	Mode of Action	References
O157:H7	*Lc. casei* strain Shirota, *L. acidophilus* YIT 0070	Yakult, Japan	Low pH, undissociated lactic acid	Growth inhibitory and bactericidal activities	[69]
O157:H7	*L. lactis*	Raw chicken meat	H_2_O_2_	Growth inhibition	[72]
O127:H6	*Li. reuteri* ATCC PTA 6475, ATCC 53608	Human, pig	Adhesins MUB, CmbA, MapA	Mucus layer binding and *E. coli* adherence decrease	[73]
O157:H7	*Li. reuteri* ATCC PTA 6475	Germfree mice	Reuterin	Decreased *E. coli* colonization, amended necrosis of the kidneys	[74]
O157:H7	*L. acidophilus* NP51	Cattle	Reuterin	Effective reduction of *E. coli* in cattle feces	[75]
EDL933	*Lc. casei* LC wt, LC CLA	Batch fermentation	Conjugated linoleic acid	Downregulation of EHEC virulence genes	[76]

**Table 2:** Antibacterial activity of lactic acid bacteria (LAB) against *Listeria monocytogenes* toxigenic producers.

Mechanism	LAB Species/Strain	Source	Agent/Action	References
Organic acids production	*Lactococcus lactis* LM0230, *Lp. plantarum, La. sakei*	Calabrian cheeses	Intracellular pH acidification for unfavorable microenvironment for non-acidophiles	[103,104]
CO_2_	Heterofermentative LAB	Foods	Anaerobic environment support; inhibition of enzyme decarboxylation; cell membrane disruption	[103]
H_2_O_2_	Heterofermentative LAB	Foods	Inactivation of essential biomolecules by superoxide anion chain reaction; activation of the lactoperoxidase system	[105,106]
Diacetyl	*Lactobacillus* sp., *Leuconostoc* sp., *P. aidilactici* CC 8081, *Streptococcus* sp.	Foods	Affects the arginine-binding proteins	[105,106,107]
Bacteriocins production	*Lactococcus lactis* subsp*. lactis, P. acidilactici, Ent. faecium, La. sakei, Li. reuteri* INIA P572, *Leu. gelidum* UAL 187, *Lc. rhamnosus* CJNU 0519	Drinks, Foods, Meats, Salads, Antimicrobial packaging	Bacteriocin synthesis: nisin, pediocin PA-1, enterocin A, sakacin A, reuterin, leucocin, rhamnocin 519	[89,105,108]
Nutrients competition	*Carnobacterium piscicola, Lactococcus piscium*	Ready-to-eat meat products	Quick uptake of nutrients by LAB; bacteriocin synthesis	[89,109,110]
Niche competition	*Li. reuteri, Li. fermentum, Lc. rhamnosus* GC mutant	Foods, probiotics	Prevent the attachment on host cells through colonization and saturation of *Lis. monocytogenes* attachment receptor	[111,112]
Reduction of *L. monocytogenes* virulence	*Li. reuteri, Li. fermentum, Lp. plantarum, Lactococcus lactis, Leu. mesenteroides, La. sakei*	Human intestinal epithelial cells (Caco-2)	Competition for adhesion receptors expressed on host cells through downregulation of virulence gens (*prfA*, *plcA*, *plcB*, *hly*, *actA*, *inlA*, *inlB*, *iap*, *luxS*)	[113,114,115]
Protection of Gastrointestinal Tract from L. monocytogenes Invasion	*Lc. casei, Li. reuteri, Lc. rhamnosus, Str. thermophilus*	Human	MUC2 and TFF3 overexpression; mucus layer integrity conservation; serum cholesterol decrease	[89,116]
Host immune response modulation	*L. bulgaricus, L. acidophilus, Lc. casei, L. salivarius, Lp. plantarum, Li. reuteri, Lc. rhamnosus, Lev. brevis, Str. thermophilus*	Human	Reduction of the pro-inflammatory cytokines (IL-8) and anti-inflammatory cytokines (IL-10) increase	[89,117]
Vaccine vector	*Lactococcus lactis*	Human	Delivery and expression of listerial antigens	[118]

**Table 3:** LAB against *C. botulinum* growth and toxin production in foods.

Strain	Metabolite	Food	References
*P. pentosaceus* 43200	Bacteriocin	Meat	[139,140,141]
*Lactococcus lactis* 11454	Nisin A	Beef	[142]
*P. acidilactici* LASC	Pediocin	Cured meat	[142]
*P. acidilactici* PO2	Pediocin	Meat	[142]
*Lp. plantarum* BN	Bacteriocin	Meat	[143]
*Streptococcus* spp.	Nisin	Cheese	[142]

**Table 4:** Antibacterial activity of Lactic acid bacteria against other bacterial toxigenic producers.

Inhibited Pathogen	LAB Species, Strain	Source	Agent	Mode of Action	References
*C. perfringens*	*L. acidophilus* CGMCC No. 1.1878, *Li. fermentum* CGMCC No. 1.2029	Chicken	Lactic acid	Bacteriostatic effect on pathogen’s growth, repression of α-toxin synthesis, α-toxin degradation by lactobacilli, *L. acidophilus* inhibits *C. perfringens* adherence to GIT epithelium	[155]
*B. cereus*	*Lactococcus lactis*, *Lactobacillus* spp.	Skim milk, fresh cheese	Organic acids, H_2_O_2_, nisin	Bactericidal effect on pathogen’s growth by leakage of cytoplasmic content of the pathogen	[166,167,168]
*B. cereus*	*L. acidophilus* LF221	Infant feces	Acidocin LF221 A and B	Bactericidal effect on pathogen’s growth	[169]
*B. cereus*	*Lactococcus lactis* C660, *Lc. paracasei* ATCC 27092	Raw milk, human	Organic acids, H_2_O_2_, nisin	Reduced adhesion of the pathogen, prevention of biofilm formation	[170]
*Pseudomonas* spp.	*Lp. plantarum, Li. fermentum, L. acidophilus, Str. thermophilus, Lactococcus lactis*	Milk	Lactic, acetic, citric acids	Reduced growth	[183]
*Ps. putida*	*Lc. paracasei* FX-6, *Lc. rhamnosus*	Milk	Organic acids	Antibacterial activity, prevention of biofilm formation	[184]
*S. aureus*	*Lactococcus lactis*	Cheese	Lantibiotics	Reduced growth by cells disruption	[185]

**Table 5:** Most harmful mycotoxins that often contaminate human food.

Type *	Genus	Foods	Clinical Picture	Molecular Mechanisms	References
Aflatoxin B_1_ (AFB_1_)	*Aspergillus*	Nuts, peanuts, maize	Extremely potent carcinogen, strongly linked with liver cancer; immunosuppression; stunted growth	Mutagenic and genotoxic effects: binds N7 of guanine; GC to TA transversions; (–) transcription, (+) oxidative stress	[191,194,195]
Ochratoxin A (OTA)	*Aspergillus*	Cereals, coffee, figs, raisins, pork kidneys	Nephrotoxic effects in all species tested; liver damage, immune suppression, and teratogenic effects in animals	(–) Phe metabolism; (–) mitochondrial ATP production; (–) tumor-suppressor gene *dmrt-1* in mice; (+) lipid peroxidation	[195,196]
Zearalenone (ZEA)	*Fusarium*	Maize, corn, other cereals	Reduced fertility, stillbirths in females; testicular atrophy and reduced spermatogenesis in males; hemato- and hepatoxic effects	ZEA-estrogen receptor complex is translocated into the nucleus which regulates the transcription of many genes	[195,197]
Fumonisins	*Fusarium*	Maize, rice, beans, beer, soybeans	Suppression of the immune response; pulmonary edema, esophageal cancer	(–) Sphingolipid synthesis; (–) mitochondrial ETC; (+) ROS generation; (+) cytotoxicity	[191,195]
Trichothecenes	*Fusarium*, *Cephalosporium*, *Myrothecium*, *Stachybotrys*, *Trichothecium*	Grains: rice, barley, oats, maize, eggs, milk, meat	Alimentary toxic aleukia (ATA): fever, diarrhea, nausea, vomiting, agranulocytosis, necrotic angina, bleeding; reduced serum levels of WBC and Ig in mice	(–) Translation; (–) mitochondrial ETC; (+) lipid peroxidation and membrane remodeling; (+) apoptosis	[191,196,198,199]
Patulin	*Penicillium*	Apples, pears, other fruits	Neurotoxic and immunotoxic effects reported in animals	As yet unknown	[195]
Citrinin	*Penicillium, Aspergillus, Monascus*	Cereals, Italian sausages	Nephrotoxic effects in all species tested; reproductive toxicity and chromosome aberrations in mice	(–) DNA and RNA synthesis; (–) microtubules assembly; (–) HSP90 multichaperone complex; (+) ROS generation	[191]
Ergot alkaloids	*Claviceps*	Various grasses and grains	Ergotism, convulsions, ataxia, gangrene, abortion	As yet unknown	[191,195]

* Trichothecenes mycotoxins are classified in groups A (T-2, HT-2); B (Deoxynivalenol, DON); C (Crotocin), and D (Verrucarins, Roridin, Satratoxins). Designations: (–), inhibits; (+), stimulates; WBC, white blood cells; Ig, immunoglobulins; ROS, reactive oxygen species; ETC, electron-transport chain; Phe, Phenylalanine.

**Table 6:** Major studies of LAB-mediated mycotoxin-related detoxification.

Target Toxin	LAB Strain	Mechanism of Action	Maximum effectiveness	References
Aflatoxin B_1_				
	*L. amylovorus* CSCC 5197 and CSCC 5160, *Lc. rhamnosus Lc1/3*	Probable adsorption on the cell surface	>50% AFB_1_ bound from solution, but reversibly	[209]
	*Lc. rhamnosus* LBGG and LC-705	None proposed	80% removal from liquid media, very rapidly	[210]
	*Lc. paracasei* LOCK 0920, *Lev. brevis* LOCK 9044, *Lp. plantarum* LOCK 0945	None proposed	39–55% decrease, depending on the initial concentration of AFB_1_	[211]
	*Lactococcus lactis*, *Lp. plantarum*	Low-molecular proteins involved, possibly bacteriocins	81% combined, 27–46% separately	[212]
	*L. kefiri* KFLM3	Toxin-binding on the cell surface	80% decrease in milk, 0% in MRS	[213]
	*Lev. brevis* NM101-1, *Lc. paracasei* ABRIINW.F58	Antifungal compounds caused 52–80% transcriptional inhibition of the *omt-A* gene, a key player in the biosynthesis of AFB_1_	90–96% reduction of the AFB_1_ production by *A. flavus* and *A. parasiticus*	[214]
	*Levilactobacillus* spp. 2QB383, *Lp. plantarum* 1QB147, 1QB314 and 3QB350	Toxin binding is assumed for the reduced amounts; no mechanism proposed for the reduced production	>50% reduced amount by inactivated strains in PPB *; >50% reduced production in YES broth at 25 °C	[215]
Ochratoxin A				
	*Str. thermophilus* T4, *L. delbrueckii* subsp*. bulgaricus* LB-51	None proposed	Complete elimination of 0.5 mg/L in milk; 36 and 26% drop with 1.0 and 1.5 mg/L	[216]
	*L. bulgaricus* 259/2 and 171/2	None proposed	Up to 94% detoxification, but very much strain-dependent	[217]
	*Lc. rhamnosus* GG, *L. acidophilus* CH-5, *L. helveticus* 8, *Lactococcus lactis* 202	Toxin binding on the cell surface is assumed, another mechanism hypothesized	60–87% decrease, rapid process but partially reversible	[218]
	*L. acidophilus* VM 20	Toxin-binding on the cell surface	96–97% decrease for 4 h	[219]
	*P. parvulus* UTAD 473	Degradation by putative peptidase	100% degradation in MRS for 7 days at 30 °C	[220]
	*Lb. kefiri KFLM3*	Toxin-binding on the cell surface	81% decrease in milk, 15% in MRS	[213]
	*Lc. rhamnosus CECT 749, Lp. plantarum* CECT 749 and CECT 288, *Lc. casei* CECT 4045, *Lc. casei* CECT 4040, *L. bulgaricus* CECT 4005	>90% degradation by proteolytic activity; very little adsorption	97–99% in MRS at pH 6.5	[221]
	*Lp. plantarum* 3QB361	Toxin-binding on cell surface assumed	~60% reduced amount by inactivated strain in PPB	[215]
Patulin				
	*Lev. brevis* 20023	Adsorption on the cell wall	65% adsorption	[222]
	*Lp. plantarum* ATCC 8014	Adsorption on the cell wall, proteins mediated	96% decrease in apple juice during 6 weeks of cold storage	[223]
	*L. kefiranofaciens* JKSP109	Adsorption on the cell wall	93% removal at pH 4.6 and 15° Brix	[224]
Deoxynivalenol				
	*Lp. plantarum* GT III	Adsorption assumed; metabolic degradation suggested	67% reduction by unviable cells (sterilized)	[225]
	*Lc. paracasei* LHZ-1	Cell wall adsorption confirmed as the major mechanism	40.7% reduction by the cell wall fraction, only 10.5 & 8.9% by SN or cellular lysate	[226]
Fumonisins				
	*Lactococcus lactis*, *L. delbrueckii*	Toxin-binding on the cell surface	75% recovery from spiked maize meal after 4 days	[227]
	*Lp. paraplantarum* CNRZ 1885, *Str. thermophilus* RAR1	Toxin binding was assumed; the role of peptidoglycan confirmed	19–37% bound FB_1_, 65–76% FB_2_, both after TCA treatment	[228]
Zearalenone				
	*Lactococcus lactis*, *L. delbrueckii*	Toxin binding assumed	68% recovery from spiked maize meal after 4 days	[227]
	*Lp. plantarum* A1	Toxin-binding on the cell surface	99% immediately, 77% after 72 h	[229]
	*Lb. kefiri* KFLM3	Toxin-binding on the cell surface	100% decrease in milk, 60% in MRS	[213]
	*Lactococcus lactis*	Surface adsorption assumed, interactions with surface proteins and intracellular uptake	90% bound in the first 20 min	[230]
	*Lp. plantarum* 3QB361	Toxin-binding on the cell surface	70–80% amount reduction by inactivated strain in PPB	[215]

* Abbreviations: PPB, Potassium Phosphate Buffer; YES, Yeast Extract Sucrose; MRS, De Man, Rogosa and Sharpe medium; AFB_1_, aflatoxin B_1_; SN, supernatant; FB_1_ and FB_2_, fumonisins B_1_ and B_2_; TCA, Trichloroacetic Acid.

**Table 7:** Detoxification of pesticides falling in food content by lactic acid bacteria (LAB).

Pesticide	LAB Species/Strain	Sample/Food	Mode of Action	References
Organochlorine				
DDT	*Lactobacillus* spp.	Cereals	Phosphotriesterase	[13]
DDT	*Streptococcus, Lactobacillus*	Ras cheese	Biodegradation	[235]
DDT	*La. sakei*	Soil	Biodegradation	[236]
Organophosphorus				
Chlorpyrifos, coumaphos, diazinon, parathion, methyl parathion	*Leuc. mesenteroides* WCP907, *Lev. brevis* WCP902, *Lp. plantarum* WCP931, *La. sakei* WCP904	Kimchi	Biodegradation	[237]
Chlorpyrifos, coumaphos, diazinon, parathion, methyl parathion	*Lev. brevis* WCP902	Kimchi	Organophosphorus hydrolase OpdB	[238]
λ-Cyhalothrin, malathion, chlorpyrifos-methyl	*Lp. plantarum* 112	Sauerkraut	Low pH	[239]
Deltamethrin, dimethoate, imidacloprid	*Lp. plantarum* 112,*Lp. plantarum* 123	Black olives	Biodegradation	[240]
Pirimiphos-methyl	*Lp. plantarum*	Wheat	Organophosphorus hydrolase, low pH	[243]
Chlorpyrifos, dichlorvos, phorate, trichlorphon	*Lp. plantarum*	Wheat dough, Chinese cabbage, Tofu	Biodegradation	[244]
Dimethoate, parathion methyl, trichlorfon	*Lp. plantarum* subsp*. plantarum* CICC 20261	Batch process	Phosphatase and Antioxydation	[245]
Phorate	*Lp. plantarum*	Corn silage	Enzyme hydrolysis	[246]
Diazinon	*L. acidophilus*	Apple juice	Enzyme hydrolysis	[247]
Diazinon, chlorpyrifos, fenitrothion, malathion	*Lev. brevis 1.0209*	Milk	Enzyme hydrolysis	[248]
Pyrethroids				
Bifenthrin	*Lp. plantarum*	Wheat flour	Enzyme hydrolysis	[241]
Beta-cypermethrin	*Lp. pentosus* 3-27	Alfalfa Silage	Enzyme hydrolysis	[242]

## Data Availability

Not applicable.
